# Comparative epigenomics reveals the impact of ruminant-specific regulatory elements on complex traits

**DOI:** 10.1186/s12915-022-01459-0

**Published:** 2022-12-08

**Authors:** Siqian Chen, Shuli Liu, Shaolei Shi, Yifan Jiang, Mingyue Cao, Yongjie Tang, Wenlong Li, Jianfeng Liu, Lingzhao Fang, Ying Yu, Shengli Zhang

**Affiliations:** 1 Key Laboratory of Animal Genetics, Breeding and Reproduction, Ministry of Agriculture and Rural Affairs & National Engineering Laboratory for Animal Breeding, College of Animal Science and Technology, China Agricultural University, Beijing, China; 2 School of Life Sciences, Westlake University, Hangzhou, China; 3 MRC Human Genetics Unit at the Institute of Genetics and Cancer, University of Edinburgh, Edinburgh, UK; 4 Center for Quantitative Genetics and Genomics (QGG), Aarhus University, Aarhus, Denmark

**Keywords:** Regulatory elements, Ruminant evolution, Liver, Epigenetic regulation, GWAS enrichment

## Abstract

**Background:**

Insights into the genetic basis of complex traits and disease in both human and livestock species have been achieved over the past decade through detection of genetic variants in genome-wide association studies (GWAS). A majority of such variants were found located in noncoding genomic regions, and though the involvement of numerous regulatory elements (REs) has been predicted across multiple tissues in domesticated animals, their evolutionary conservation and effects on complex traits have not been fully elucidated, particularly in ruminants. Here, we systematically analyzed 137 epigenomic and transcriptomic datasets of six mammals, including cattle, sheep, goats, pigs, mice, and humans, and then integrated them with large-scale GWAS of complex traits.

**Results:**

Using 40 ChIP-seq datasets of H3K4me3 and H3K27ac, we detected 68,479, 58,562, 63,273, 97,244, 111,881, and 87,049 REs in the liver of cattle, sheep, goats, pigs, humans and mice, respectively. We then systematically characterized the dynamic functional landscapes of these REs by integrating multi-omics datasets, including gene expression, chromatin accessibility, and DNA methylation. We identified a core set (*n* = 6359) of ruminant-specific REs that are involved in liver development, metabolism, and immune processes. Genes with more complex cis-REs exhibited higher gene expression levels and stronger conservation across species. Furthermore, we integrated expression quantitative trait loci (eQTLs) and GWAS from 44 and 52 complex traits/diseases in cattle and humans, respectively. These results demonstrated that REs with different degrees of evolutionary conservation across species exhibited distinct enrichments for GWAS signals of complex traits.

**Conclusions:**

We systematically annotated genome-wide functional REs in liver across six mammals and demonstrated the evolution of REs and their associations with transcriptional output and conservation. Detecting lineage-specific REs allows us to decipher the evolutionary and genetic basis of complex phenotypes in livestock and humans, which may benefit the discovery of potential biomedical models for functional variants and genes of specific human diseases.

**Supplementary Information:**

The online version contains supplementary material available at 10.1186/s12915-022-01459-0.

## Background

Over the past decade, genome-wide association studies (GWAS) have successfully discovered hundreds of thousands of genetic variants associated with complex traits and diseases in both human and livestock species [1–3]. As the majority of these variants are located in noncoding regions [4], it is challenging to understand how they impact complex phenotypes. Previous studies have illustrated that trait-associated variants are significantly enriched in regulatory regions, such as promoters and enhancers, in well-studied species (e.g., humans and mice) [5, 6]. Currently, global efforts such as the Functional Annotation of Animal Genomes (FAANG) initiative and the Farm animal Genotype-Tissue Expression (FarmGTEx) project are working to uncover basic knowledge of genomic function and regulation in livestock species [7–9]. However, a comprehensive atlas of regulatory elements (REs) is still lacking for most livestock species, which limits our understanding of the functional biology of species evolution and restricts the genetic improvement of complex traits in livestock. As abundant terrestrial herbivores [10], ruminants, such as cattle, sheep, and goats, have a unique history of species differentiation and play an important economic role in modern animal husbandry. Therefore, a comprehensive comparison of REs between major ruminants and other species will provide novel insights into functional genome evolution specific to ruminants. Moreover, it will allow us to explore the genetic basis underlying complex traits of economic value in these farm animal species.

The emergence of cross-species comparative epigenomics has provided a new method for both elucidating genomic evolution and identifying potential functional noncoding variants associated with complex traits and diseases [11]. By comparing the chromatin landscape of primary aortic endothelial cells isolated during the acute NF-κB response among humans, mice, and cattle, Alizada et al. found that inflammatory- and cardiovascular-associated genetic variants discovered by GWAS were significantly enriched in the species-conserved regulatory regions nearby NF-κB target genes [12]. In addition, by cross-species mapping of epigenomic marks, Liu et al. found that the genetic control of immune and reproductive traits is conserved to a certain degree between humans and cattle [13]. These findings indicate that evolutionarily conserved REs play key roles in shaping complex phenotypes across species [14, 15]. Although previous studies have investigated the evolution of the transcriptome (e.g., long noncoding RNAs) in ruminants [8, 16], a comprehensive comparison of epigenetic regulation and its potential impacts on other molecular phenotypes and complex traits is still lacking.

Here, by using the liver as a representative tissue, we systematically detected and functionally characterized the epigenomic landscapes and explored the dynamics of REs across three ruminant species (i.e., cattle, sheep, and goat) and three non-ruminant species (i.e., pig, mouse, and human). We annotated an average of 81,081 REs (17,154 and 63,927 promoters and enhancers, respectively) across six species by integrating 137 multi-omics datasets, including epigenetic data such as histone modifications, gene expression, chromatin accessibility, and DNA methylation (Additional file 1: Fig. S1). By detecting lineage-specific REs and associating them with expression quantitative traits loci (eQTLs) and large-scale GWAS datasets from 44 and 52 complex traits in cattle and humans, respectively, we further explored how comparative epigenomics across species could help us understand the evolutionary and genetic mechanism of complex phenotypes. Overall, our study provides a valuable resource for REs in ruminants and highlights the key roles of conserved functional elements in complex traits in both human and livestock species.

## Results

### Overview of multi-omics datasets

**Fig. 1:**
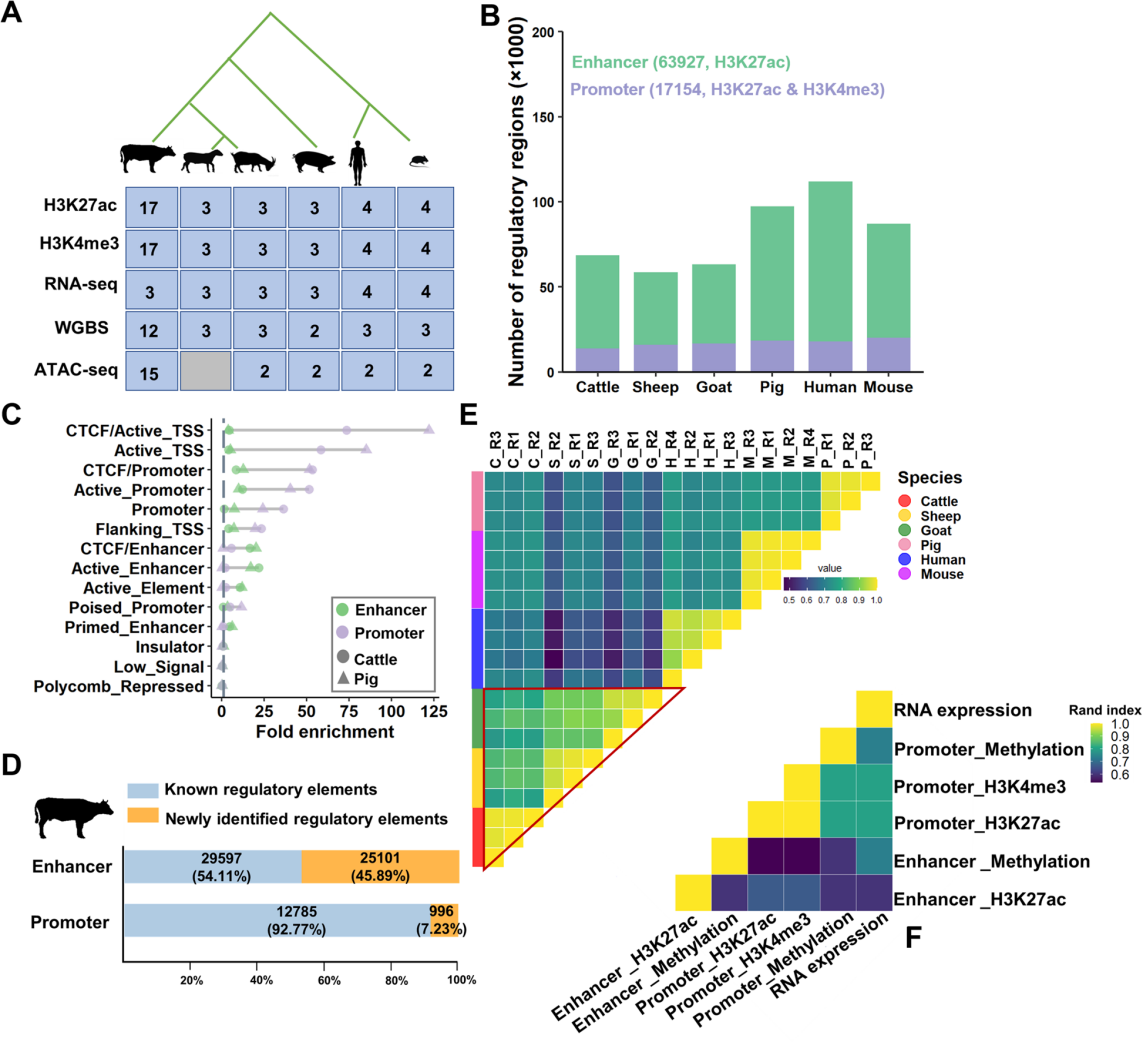
Summary and characterization of 137 epigenetic and gene expression data in six mammals. **A** Datasets analyzed by this study. **B** The number of regulatory regions (promoters and enhancers) identified in the liver of each species. **C** Fold enrichments of regulatory elements (REs) for 14 chromatin states previously predicted in cattle and pig liver [19]. These chromatin states mainly represented enhancers (CTCF/Enhancer, Active_Enhancer, and Primed_Enhancer), promoters (CTCF/Promoter, Active_Promoter, Promoter, Poised_Promoter), repressed regions (Insulator, Low_Signal, and Polycomb_Repressed), open regions (Active_Element), and TSS-proximal regions (CTCF/TSS, Active_TSS, and Flanking TSS). **D** The percentages of REs overlapped with public data in cattle liver (blue) and newly annotated in this study (orange). **E** The sample clustering based on pairwise Spearman correlation of gene expression. **F** Similarity of sample clustering patterns across different omics data types using Rand index

To study the epigenomic changes during ruminant evolution, we performed chromatin immunoprecipitation sequencing (ChIP-seq) for H3K27ac and H3K4me3 in the liver of cattle, sheep, and goats (Fig. 1A). In total, we generated 17 ChIP-seq datasets, and each species had three biological replicates. We also generated nine RNA sequencing (RNA-seq) datasets and nine whole genome bisulfite sequencing (WGBS) datasets in the same liver samples. We further retrieved a total of 41 public datasets including ChIP-seq datasets for H3K27ac and H3K4me3, WGBS datasets, and RNA-seq datasets, of three non-ruminant (i.e., pigs, humans, and mice) livers. Each species had at least two biological replicates (Additional file 2: Table S1A-C) [17, 18]. We have processed all the data using the same data analysis pipeline to make human, mouse, and pig datasets (previously generated) comparable to those (newly generated) of three ruminants. Furthermore, we also collected datasets from seven other cattle tissues to investigate the dynamic epigenetic landscape across tissues [19]. Overall, we uniformly analyzed 35 new genome-wide omics datasets from three ruminant livers and integrated them with 102 previously published datasets. We obtained over 25 billion mapped reads with an average mapping rate of 91.24% after filtering low-quality reads (Fig. 1A and Additional file 2: Table S1A-D).

Through signal saturation analysis, we found that 20 million reads were required to reach the saturation of consistent peak detection for H3K27ac and H3K4me3 in single-end ChIP-seq samples, while 37.5 million reads were required for paired-end ChIP-seq samples (Additional file 1: Fig. S2A and S2B). We detected 66,000–108,000 (mean = 83,338) H3K27ac-enriched regions and 18,000–29,000 (mean = 23,576) H3K4me3-enriched regions in liver (*q* < 0.01, Additional file 1: Fig. S2C and S2D). Furthermore, we defined two categories of REs, (1) promoters, which were simultaneously marked by H3K4me3 and H3K27ac, and (2) enhancers, which were only enriched for H3K27ac. We identified an average of 81,081 REs per species, including 63,927 enhancers and 17,154 promoters (Fig. 1B and Additional file 3: Table S2). Moreover, we found that enhancers exhibited higher tissue specificity compared to promoters. For example, we found that a majority of (85.94%) enhancers in the liver exhibited tissue specificity, while only 22.92% of promoters did (Additional file 1: Fig. S2E). We observed that 78.09% of all promoters were located around (distance ≤ 5kb) transcriptional start sites (TSSs), whereas the majority (77.88%) of enhancers were distal to TSSs (distance > 5kb) (Additional file 1: Fig. S3A), which was consistent with previous findings [17]. We calculated the enrichment fold of REs for 14 chromatin states previously predicted by ChromHMM [19] and observed that these REs were significantly enriched for the corresponding chromatin states (Fig. 1C and Additional file 1: Fig. S3B and S3C). For instance, enhancers were significantly enriched for “Active_Enhancer” and “CTCF/Enhancer” (enrichment fold = 21.78 and 16.61, respectively). We further validated that over 70% and 45% of our newly detected promoters and enhancers overlapped with REs identified using publicly available datasets in the liver (Fig. 1D and Additional file 1: Fig. S3D and S3E) of cattle, pigs, and mice [17, 19, 20]. Overall, these results indicate the high reliability of the REs identified in this study. Notably, we also newly identified REs in the liver that had not been annotated in previous studies. For example, 7.23% and 45.89% of all cattle promoters and enhancers were newly identified in this study, respectively (Fig. 1D).

To evaluate the evolution of epigenomic marks and gene expression across these six species, we performed hierarchical clustering based on epigenomic mark signal intensities and expression levels of 9796 one-to-one orthologous genes. As expected, we observed that the three ruminants were clustered together, consistent across all six omics data types (Fig. 1E and Additional file 1: Fig. S4). This relationship pattern across species was consistent in terms of gene expression and epigenomic marks, which were measured by the pairwise Rand index (Fig. 1F). These observations reflected the consistent effects of epigenomic marks, gene expression, and genome during species divergence.

### Co-evolution of epigenomic regulatory and gene expression

**Fig. 2:**
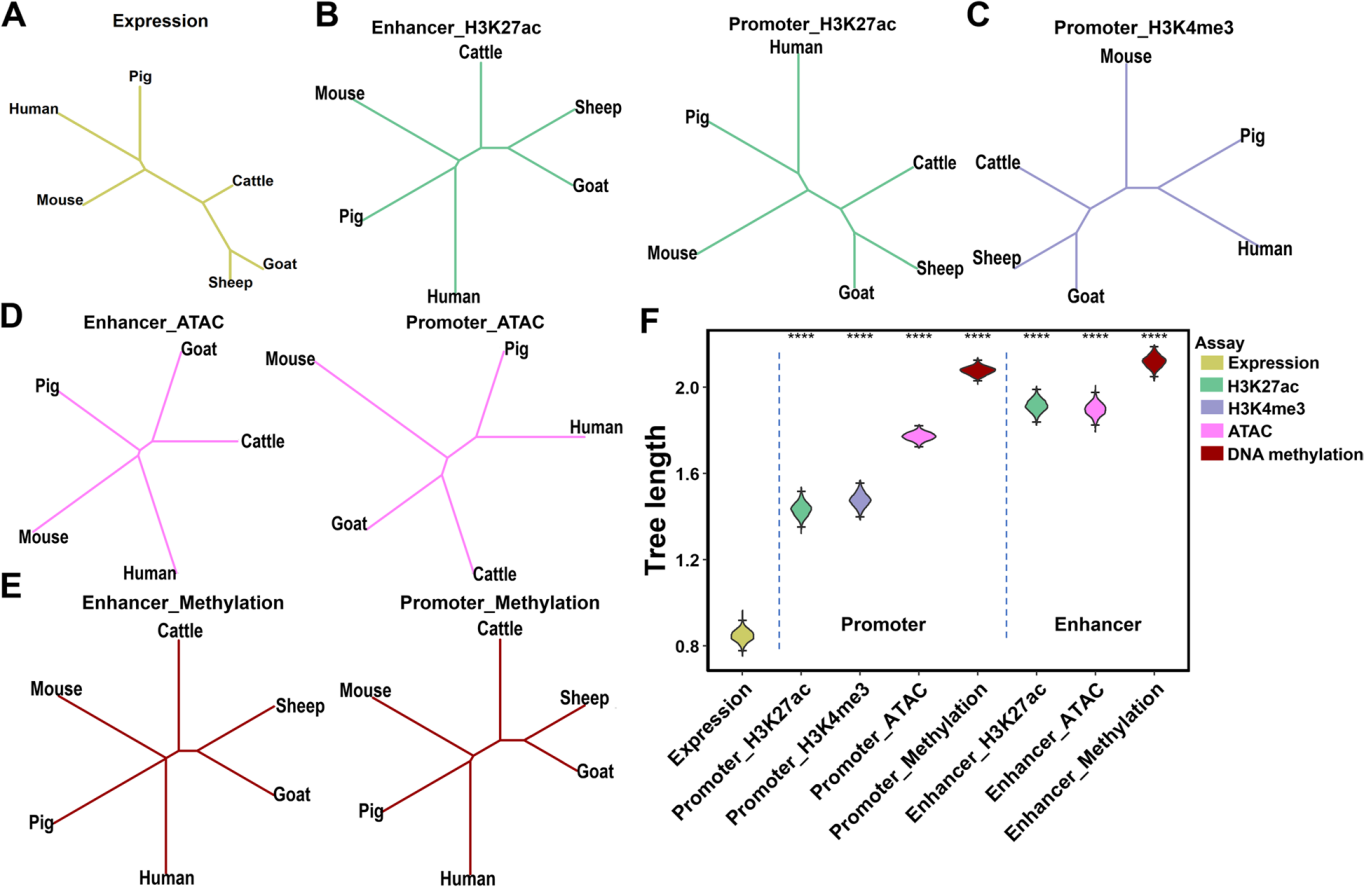
Evolutionary changes of regulatory elements (REs) and gene expression across mammals. Phylogenetic trees were built using the neighbor-joining method for gene expression of 9796 one-to-one orthologous genes (**A**), H3K27ac signals (**B**), H3K4me3 signals (**C**), chromatin accessibility (**D**), and DNA methylation level (**E**) in REs (enhancers and promoters) of orthologous genes. **F** Comparisons of total branch lengths of the phylogenetic trees across five omics data types using 1000 bootstrapping test. “****” indicates *P* < 0.0001

To obtain a global view of the evolution of REs and gene expression, we constructed phylogenetic trees for five distinct omics data types, including ChIP-seq for H3K27ac and H3K4me3, RNA-seq (gene expression), WGBS (DNA methylation), and ATAC (chromatin accessibility) (Fig. 2A–E). All eight phylogenetic trees were in agreement with the known genome phylogeny across six species [21]. We observed two major mammalian lineages (ruminants and non-ruminants), followed by the separation among the ruminant lineages (Bovidae and Caprinae). This implies co-evolution and interplay among different functional genome elements and DNA sequences during mammalian evolution.

According to bootstrapping analysis, the total length of the phylogenetic trees varied widely across these omics data types (Fig. 2F), reflecting their differences in evolution rates. Notably, the branch of the gene expression tree was significantly (*P*<0.0001) shorter than those of another seven epigenomic phylogenetic trees, indicating that gene expression levels were more conserved than their regulatory elements during mammalian evolution. Furthermore, promoters were highly conserved compared to distal enhancers [17, 22]. Compared to histone modifications (H3K27ac and H3K4me3) and chromatin accessibility, DNA methylation in REs evolved faster (Fig. 2F) [23].

### Dynamic landscape of hepatic REs during ruminant evolution

**Fig. 3:**
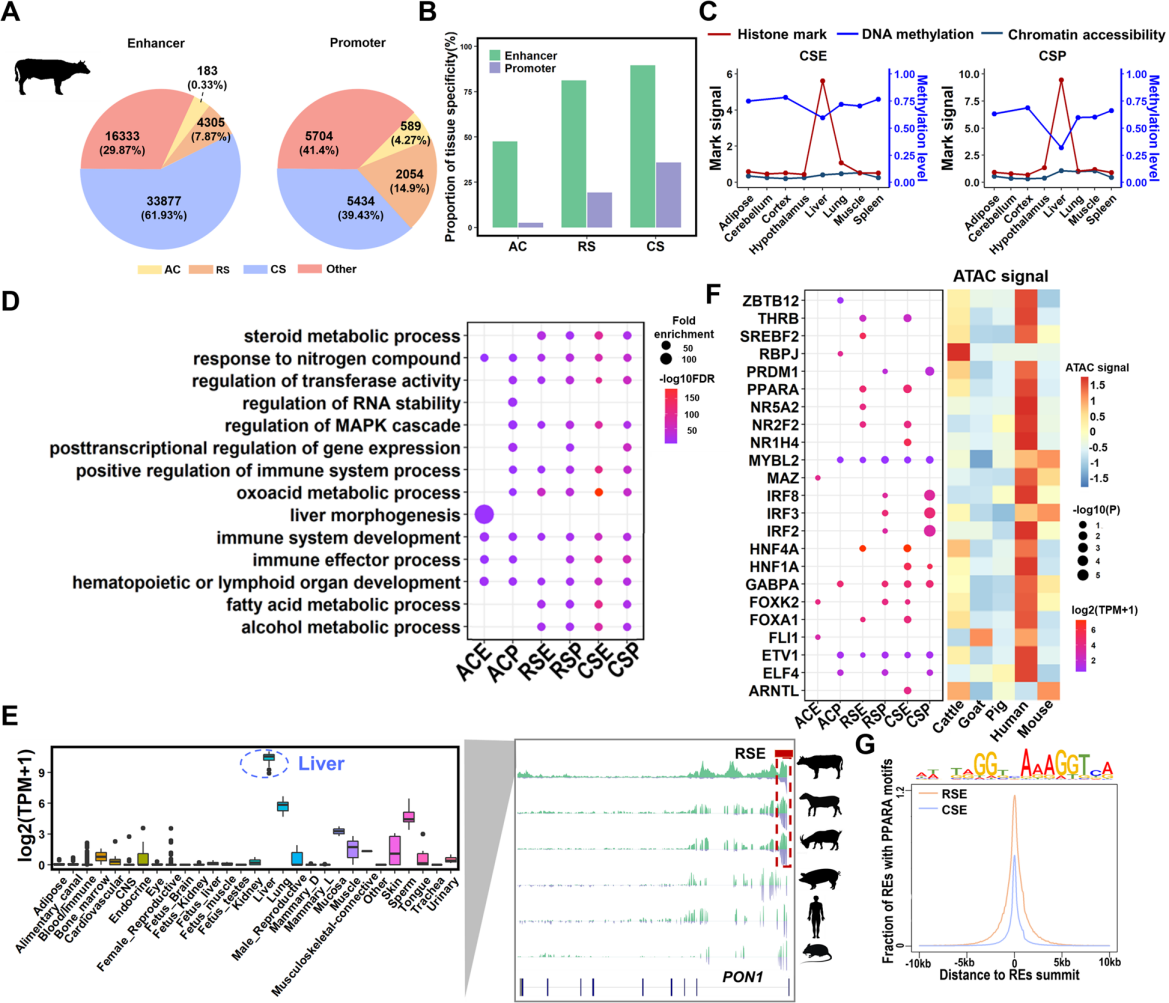
The dynamic of regulatory elements (REs) in the liver during ruminant evolution. **A** The fractions of REs that are highly conserved (AC), ruminant-specific (RS), and cattle-specific (CS) in cattle. **B** Specificity of liver REs determined by comparing to the other seven cattle tissues-adipose, cortex, cerebellum, hypothalamus, lung, spleen, and muscle. **C** Histone mark signals (H3K4me3 for cattle-specific promoters (CSP), H3K27ac for cattle-specific enhancers (CSE)), DNA methylation levels, and chromatin accessibility of tissue-specific CSE and CSP across eight cattle tissues. **D** GREAT Gene Ontology (GO) terms enrichment for six lineage-specific REs (FDR <0.01). **E** H3K27ac and H3K4me3 ChIP-seq profiles of *PON1* gene (right) for six species, cattle, sheep, goat, pig, human, and mouse, at the loci of ruminant-specific enhancers (RSE). The expression levels of the *PON1* gene (left) in the 28 organ systems of cattle. TPM, transcripts per kilobase million. **F** Transcription factor (TF) motifs enriched in six lineage-specific REs. The bubble plot shows the enrichment of TF motifs in six lineage-specific REs, and the heatmap shows the chromatin accessibility of TF promoters across five mammals. **G** The fraction of RE peaks harboring canonical PPARA motif (JASPAR – MA1148.1) as a function of distance from RE peaks to the summits

To further investigate the epigenomic molecular mechanisms underlying ruminant evolution, particularly in cattle, we divided cattle REs into three main categories using cattle as the “anchor” species based on their absence/presence among six species, i.e., highly conserved (AC), ruminant-specific (RS), and cattle-specific (CS) REs (Additional file 1: Fig. S5A). We identified 772 AC-REs across six mammals, including 183 enhancers and 589 promoters (Fig. 3A and Additional file 1: Fig. S5B-F). Consistent with the previous observation that promoters had a high conservation level than enhancers in the liver across primates [17], we observed that ~4.27% of promoters were AC-Promoters, whereas only ~0.33% of enhancers were AC-Enhancers. By comparing with the REs detected in another seven bovine tissues [19], we observed that REs in the liver with higher lineage-specificity exhibited higher tissue specificity, which was consistent for both enhancers and promoters. For instance, by comparing with the other seven tissues, we found that 47.54% and 89.61% of highly conserved enhancers (AC-Enhancers) and cattle-specific enhancers (CS-Enhancers) were specific in the liver, respectively (Fig. 3B). In general, we found that enhancers are more tissue-specific than promoters, consistent with the hypothesis that enhancers are important regulators of tissue-specific gene expression and are highly related to the function of the respective tissues [15, 24]. In addition, liver-specific REs exhibited hypomethylation and high expression of their target genes specifically in the liver (Fig. 3C and Additional file 1: Fig. S6 and S7) [25, 26].

Based on gene ontology (GO) enrichment analysis, we found that the putative target genes of different lineage-specific REs were significantly (FDR<0.01) enriched for distinct biological processes (Fig. 3D and Additional file 4: Table S3). Genes linked to AC-REs were significantly involved in fundamental and developmental biological functions (e.g., regulation of RNA stability and liver morphogenesis), whereas those linked to RS- and CS-REs were enriched in metabolic and immune processes (e.g., steroid metabolic processes and positive regulation of immune system processes). For instance, a liver-specific ruminant-specific enhancer (RS-Enhancer) located within the *PON1* gene was shared across three ruminants (Fig. 3E), which plays an important role in inhibiting low-density lipoprotein oxidation and has rapidly evolved in the ancestor of ruminants [27, 28]. Moreover, *PON1* had high expression levels specifically in adult livers of cattle (Fig. 3E) [25]. To investigate potential transcription factors (TFs) change during ruminant evolution, we performed TF motif enrichment analysis for lineage-specific REs. The enriched TFs had similar biological functions as genes linked to the corresponding lineage-specific REs and exhibited matched expression levels and chromatin accessibility across species (Fig. 3F and Additional file 5: Table S4). For example, *RBPJ* and *MAZ*, which are involved in intrahepatic bile duct development and transcription initiation [29–32], were significantly enriched in AC-REs, whereas *HNF4A*, *PPARA*, and *FOKA1* which are associated with hepatocyte proliferation, glucagon biosynthesis, and lipid metabolism [33–35] were enriched in RS-Enhancers and CS-Enhancers. We further observed that RS-Enhancers contained more liver-related motifs than CS-Enhancers, as exemplified by the fraction of motif for the PPARA transcription factor (Fig. 3G). Moreover, we found specific enrichment of *IRF* motif families in ruminant-specific promoters (RS-Promoters) and cattle-specific promoters (CS-Promoters). Overall, we provide a valuable resource for lineage-specific REs, which play an important role in multiple fat metabolism processes, immune function, and hepatocyte development.

### The number of REs contributes to gene expression conservation across species

**Fig. 4:**
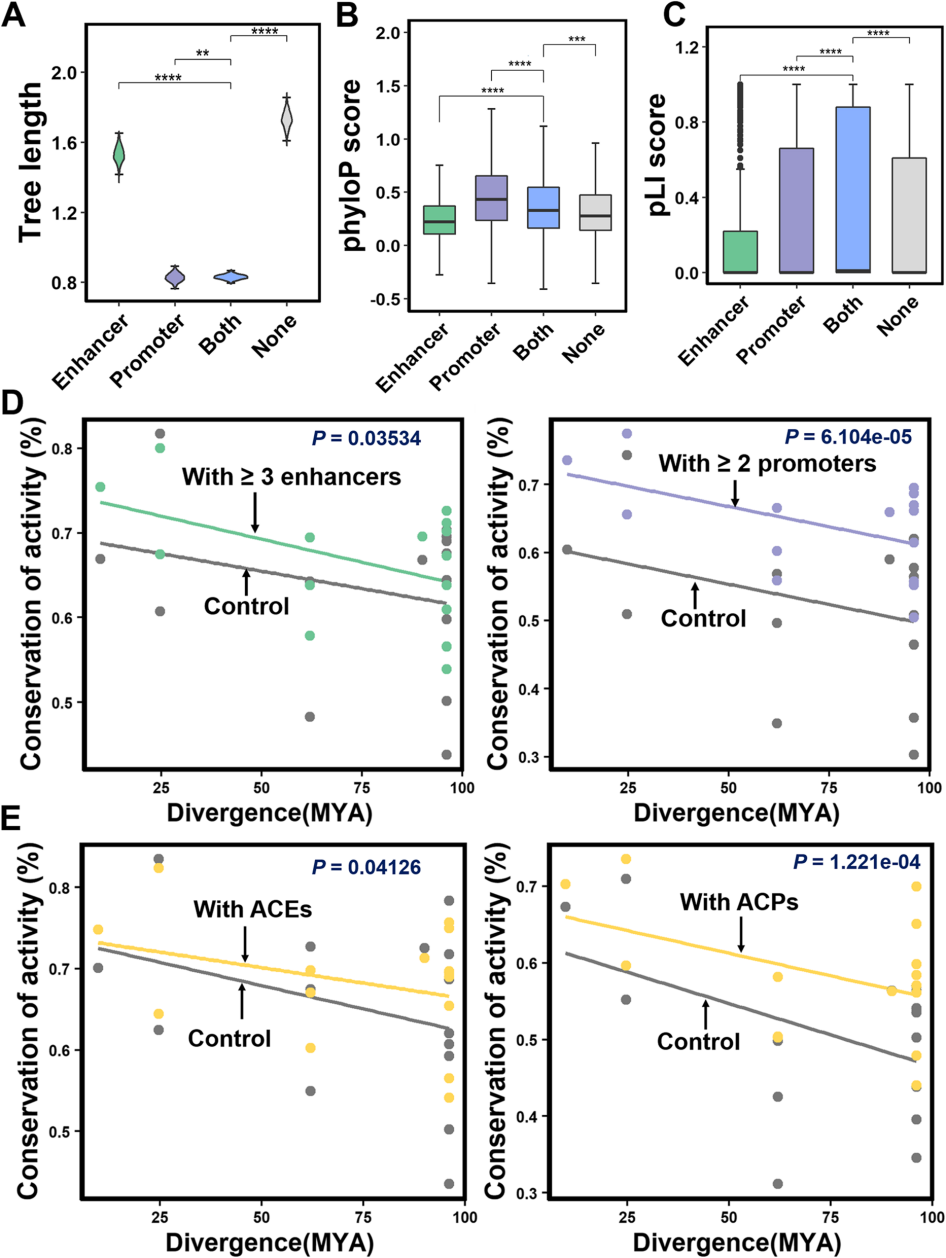
Regulatory elements (REs) drive interspecies transcriptional conservation during evolution. **A** Comparison of total branch lengths for the phylogenetic trees across four categories of one-to-one orthologues genes based on the 1000 bootstrapping test. Enhancer, Promoter, Both, and None indicate genes with only enhancers, only promoters, both enhancers, and promoters, without REs, respectively. Box plots showing the median phastCons scores (**B**) and pLI score (**C**) for four categories of one-to-one orthologues genes. **D** The number of associated enhancers and promoters contributes to interspecies transcriptional stability. **E** The association of gene stability and conserved REs, including highly conserved enhancers (ACE) and highly conserved promoters (ACP). “**” indicates *P* < 0.01; “***” indicates *P* < 0.001; “****” indicates *P* < 0.0001

To detect how the evolution of enhancers and promoters regulates interspecies changes in gene expression during ruminant evolution, we generated RNA-seq datasets to quantify gene expression levels from a total of six species. We then grouped genes into four categories: (1) genes with both enhancers and promoters (Both); (2) genes with only promoters (Promoter); (3) genes with only enhancers (Enhancer), and (4) genes with none of the defined REs (None). Firstly, we found that “Both” and “Promoter” genes had the highest conservation, followed by “Enhancer” genes, while “None” genes showed the lowest conservation (Fig. 4A and Additional file 1: Fig. S8). The phyloP sequence conservation score (Fig. 4B) and probability of loss-of-function intolerance (pLI) score (Fig. 4C) of “Both” and “Promoter” genes were also higher than that of the other two gene sets. Next, we tested the relationship between the number of REs and gene expression levels. Consistent with previous studies [36, 37], genes with more REs tended to have higher gene expression levels (Additional file 1: Fig. S9 and S10), illustrating that the majority of the REs identified in this study showed an additive effect on gene expression levels. Moreover, we observed that the number of REs of a gene could also influence interspecies conservation of gene expression. Genes associated with multiple REs (i.e., enhancers ≥ 3; promoters ≥ 2) exhibited significant transcriptional conservation, compared to those with no or fewer REs (i.e., enhancers ≤ 2; promoters ≤ 1) (Wilcoxon signed-rank test: enhancers *P* = 0.0354 and promoters *P* = 6.104e−05; Fig. 4D). Overall, these results further support that the redundancy of REs may contribute to buffering in transcriptional evolution and regulatory innovation [36–38].

To determine whether the conserved REs contribute to the evolutionary stability of gene expression across species, we compared the evolutionary stability of genes with similar expression levels, but differing in the absence or presence of conserved REs. Genes with AC-REs exhibited higher conservation than those without (Wilcoxon signed-rank test: AC-Enhancers *P* = 0.04126 and AC-Promoters *P* = 1.221e−04, respectively; Fig. 4E). These results indicate that conserved REs contribute to the evolutionary conservation of gene expression.

### REs are enriched for genomic variants and QTLs of complex traits

**Fig. 5:**
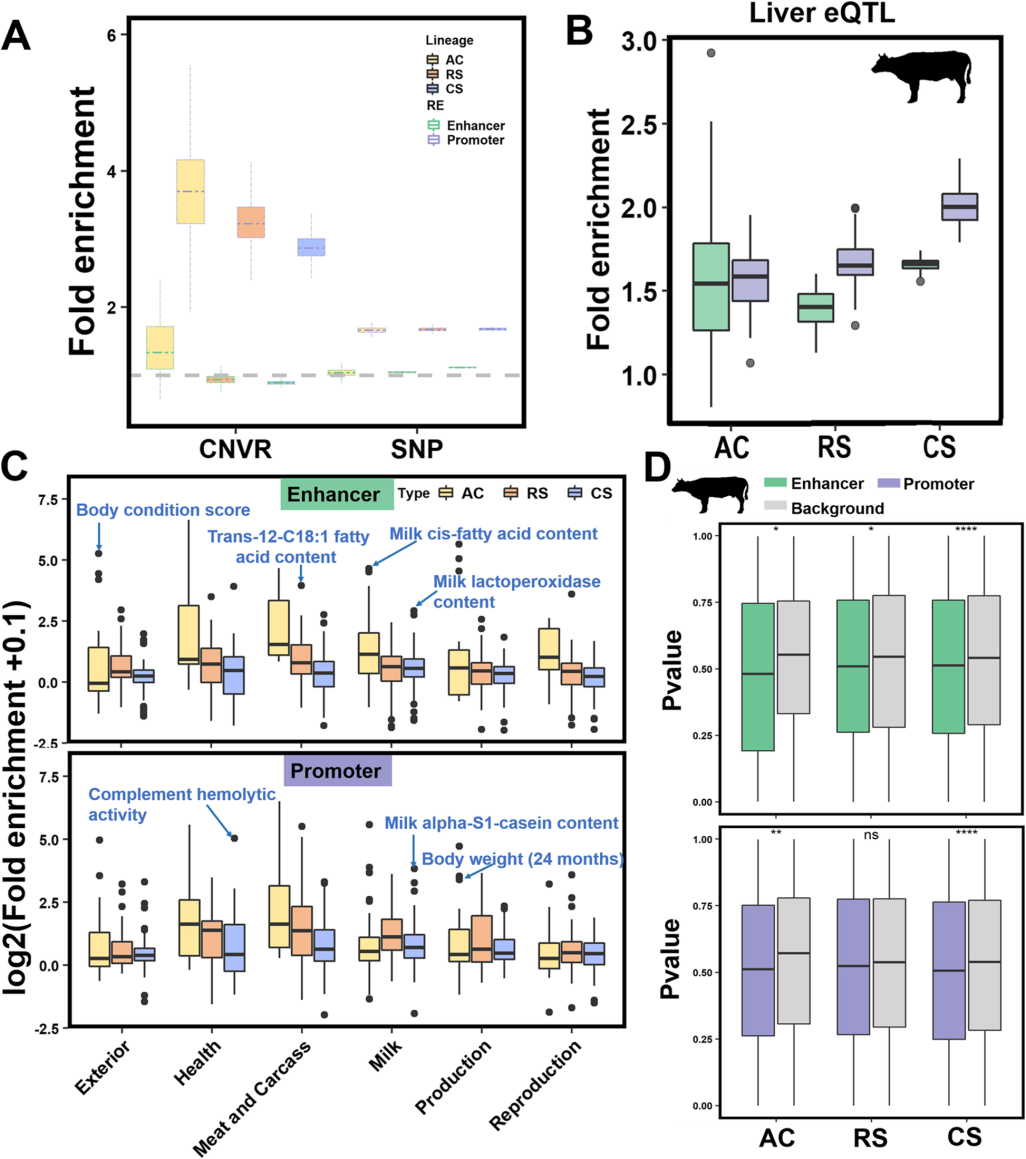
Regulatory elements (REs) are enriched for genomic variants of gene expression and complex traits. The fold enrichment of REs for genomic variants [39] (**A**), expression QTLs (eQTLs) detected from cattle liver [9] (**B**), and six categories of QTLs (including 489 complex traits) downloaded from the cattle QTL database [43] (**C**), based on 1000 bootstrapping test. **D** The *P* values of SNPs inside and outside of REs from GWAS summary datasets for protein percentage in cattle [3]. “*” indicates *P* < 0.05; “**” indicates *P* < 0.01; “****” indicates *P* < 0.0001; “ns” indicates *P* >0.05

To further investigate the relationship between characterized REs and genomic mutations, we calculated the enrichment of REs for single-nucleotide polymorphisms (SNPs) and copy number variable regions (CNVRs) in cattle obtained from the NCBI database of SNP (dbSNP) and Animal Omics Database (AOD) [39]. We found that promoters had higher enrichment for CNVRs and SNPs than enhancers, while lineage-specific REs had a lower enrichment for CNVRs than conserved ones (Fig. 5A). These observations were consistent with previous CNV annotation in humans that CNVRs prefer to be near promoters and away from enhancers [40, 41]. Moreover, the density of SNPs increased in the proximity of REs (Additional file 1: Fig. S11A), particularly for CS-Promoters. We performed enrichment analyses using eQTLs in cattle livers with lineage-specific REs [9]. All six types of REs were significantly enriched for eQTLs, with the enrichment fold ranging from 1.41 to 2.03 (*P* < 0.05, 1000 bootstrapping test). These eQTLs exhibited higher enrichment in promoters of matching lineage-specific types than in enhancers, as previously reported in humans (Fig. 5B) [42]. This result indicates that lineage-specific REs may have played important roles in regulating gene expression during mammalian evolution.

By overlapping REs with QTLs of 489 different cattle traits from the cattle QTL database (QTLdb), we observed that all six types of REs showed enrichments for QTLs of six trait categories (Fig. 5C and Additional file 6: Table S5A-F). The top enriched complex traits were associated with liver function, such as milk alpha-S1-casein content and milk cis-fatty acid content. Body condition score and body weight (24 months) were enriched for AC-Promoters, which was consistent with a previous study on beef cattle that suggested the liver is the relevant tissue of feed efficiency and is associated with daily weight gain through regulating metabolism processes [44]. Moreover, SNPs inside REs showed significantly stronger associations (as represented by the *P*-value) than those outside REs for both cattle and human traits, such as protein percentage in cattle and alkaline phosphatase in humans (Fig. 5D and Additional file 1: Fig. S11B). In summary, these observations further illustrate that lineage-specific REs are hotspots of causative mutations for complex traits.

### Enrichment analysis of REs for GWAS signals of complex traits in cattle and humans

**Fig. 6:**
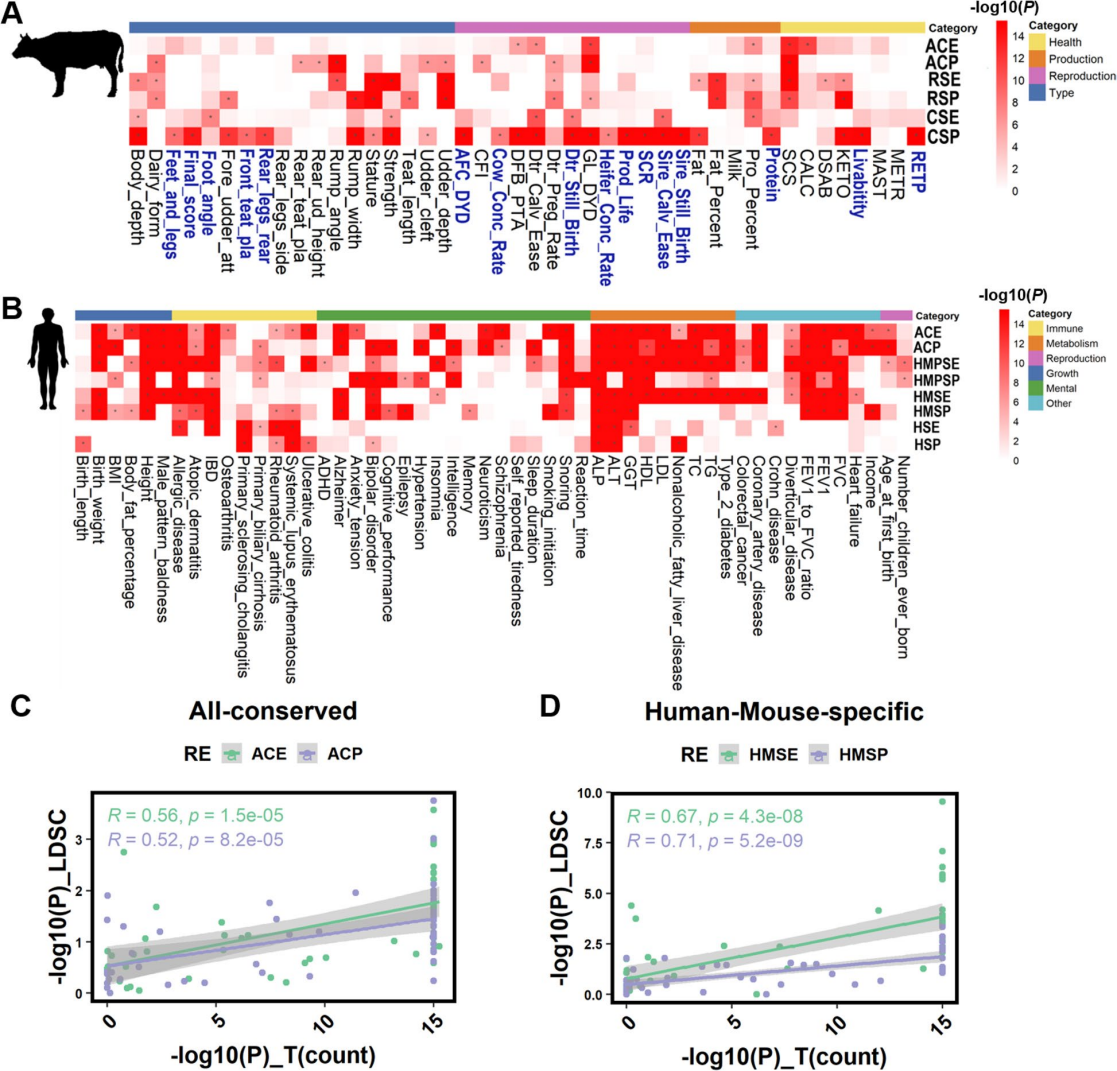
GWAS signal enrichment analysis of regulatory elements (REs) for complex traits and diseases. GWAS signals enrichment analysis of the characterized REs for 44 and 52 complex traits in cattle (**A**) and humans (**B**), respectively. “Blue” represents traits that were only significantly enriched in cattle-specific REs (CS-REs) in **A**. “*” indicates *P* < 1.0e−5. ACE, ACP, RSE, RSP, CSE, CSP, HMPSP, HMPSE, HSE, and HSP stand for all-conserved enhancers, all-conserved promoters, ruminant-specific enhancers, ruminant-specific promoters, cattle-specific enhancers, cattle-specific promoters, human-pig-mouse-specific enhancers, human-pig-mouse-specific promoters, human-pig-specific enhancers, human-pig-specific promoters, human-specific enhancers, human-specific promoters, respectively. **C, D** Pearson’s correlations of enrichment degrees (−log_10_*P*) across 52 human traits calculated by using the stratified linkage disequilibrium score regression (LDSC) and the count-based marker-set test

To further investigate how the conservation of REs shapes the genetics of complex traits in cattle and humans, we conducted GWAS signal enrichment analyses of the characterized REs using GWAS summary statistics from 44 and 52 complex traits in cattle and humans, respectively (Fig. 6A, B, Additional file 7: Table S6, and Additional file 8: Table S7). For human traits, we also conducted the heritability enrichment analysis using the stratified linkage disequilibrium score regression (LDSC) [45], and found that the results from LDSC were significantly and positively correlated with those from the count-based test (Fig. 6C, D). We did not conduct the similar analysis as the matched linkage disequilibrium (LD) and minor allele frequency (MAF) were not available for the Holstein population being analyzed. It would be of interest to consider the LD and genetic relatedness of individuals when conducting the heritability enrichment analysis in livestock, once the individual genotypes and phenotypes are available.

**Fig. 7:**
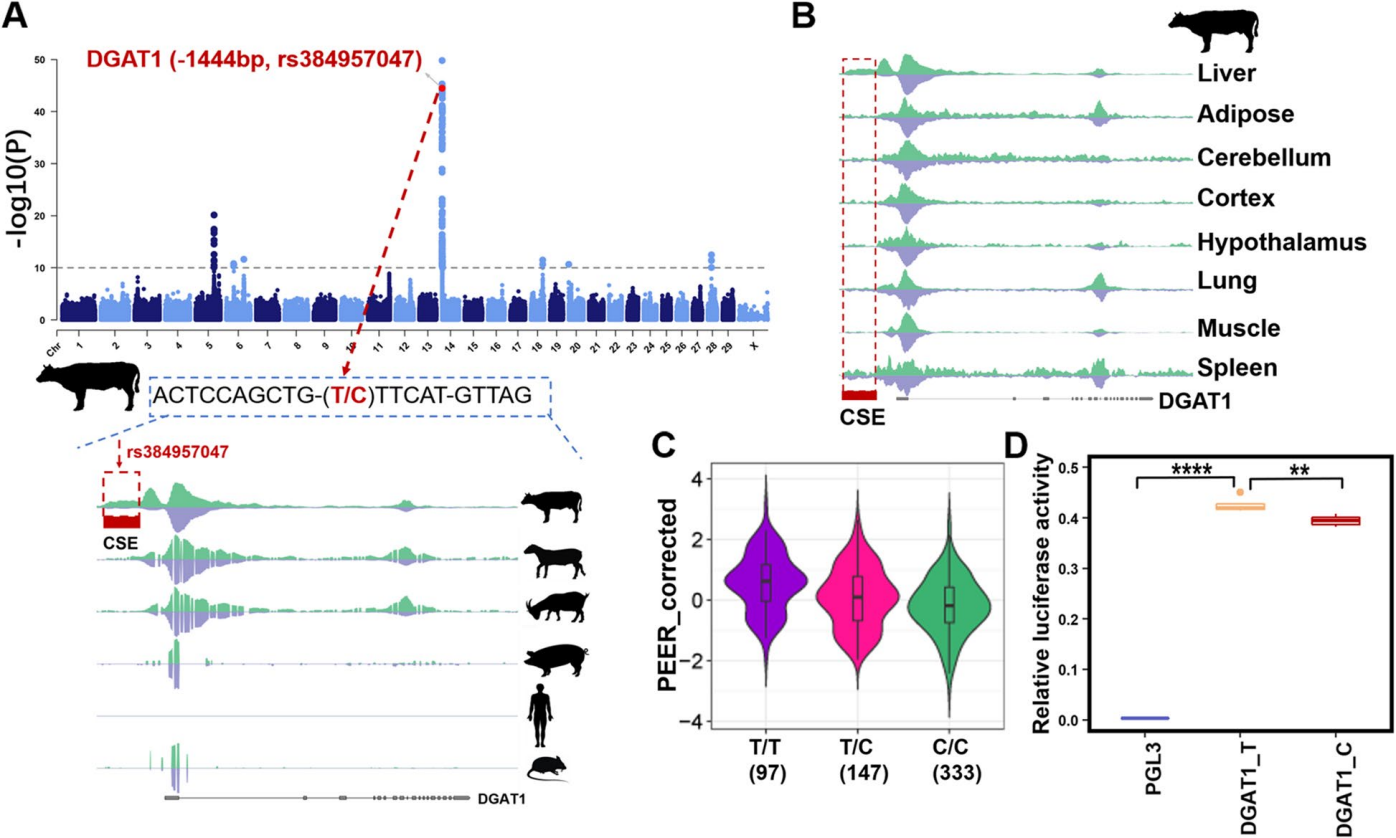
A cattle-specific enhancer of *DGAT1* was associated with protein percentage in cattle. **A** The top panel is the Manhattan plot of protein percentage in cattle [3]. The bottom panel shows H3K27ac (green) and H3K4me3 (purple) profiles within the *DGAT1* locus across six species. **B** H3K27ac (green) and H3K4me3 (purple) profiles within the *DGAT1* locus across eight tissues in cattle. **C** The PEER-corrected expression level of *DGAT1* is significantly associated with three genotypes of *rs384957047* in cattle liver [9]. **D** The relative luciferase activity of the recombinant plasmids constructed with DGAT1_T and DGAT1_C of *rs384957047*. “**” indicates *P* < 0.01; “***” indicates *P* < 0.001

In general, CS-/RS-REs showed a higher enrichment for many cattle complex traits than conserved ones, whereas the opposite trends were observed for human complex traits, where human-specific REs showed a lower enrichment for human complex traits than conserved ones. Notably, we found that highly conserved REs were enriched for similar complex traits in humans and cattle. For instance, AC-Promoters were significantly enriched for somatic cell score (SCS) (*P* < 1.00e−15) in cattle and primary biliary cirrhosis (*P* < 3.51e−08) in humans (Fig. 6A, B). This was consistent with a previous study, which reported that conserved regulatory regions enriched for SNPs associated with inflammatory traits in humans [12]. Furthermore, we noted that GWAS signals for some complex traits were regulated by both AC- and RS-/CS- specific REs (Fig. 6A), such as protein percentage in cattle. These results suggest that for some complex traits, cross-species comparison of the functional genome may help us interpret the biological mechanisms underlying complex traits, and then enhance genetic improvement in livestock species. We also found that GWAS signals for 16 out of 44 complex traits were only significantly enriched in CS-REs, including five body type traits, eight reproductive traits, one production trait, and two healthy traits (Fig. 6A). This may be due to the strong artificial selection of complex traits of economic value in cattle. Interestingly, we noted that lineage-specific enhancers were enriched for GWAS signals of both protein percentage and ketosis in cattle (Fig. 6A). For instance, a liver-specific CS-Enhancer within *DGAT1* harbored risk SNPs associated with protein percentage (*rs384957047, P* < 1.80e−18), which is located in the upstream of *DGAT1* [3, 46] (Fig. 7A, B). *DGAT1* is necessary for triacylglycerol synthesis and has been reported to be associated with milk production, milk fat composition, metabolizability, and N efficiency in cattle [47–49]. The expression level of *DGAT1* was significantly regulated by *rs384957047* in cattle liver, which is a fine-mapped *cis*-eQTL (*P* < 1.14e−11, Fig. 7C). Moreover, transcription factor binding prediction analysis showed that TFs were changed before and after the SNP *rs384957047* mutation in the upstream of *DGAT1* (Additional file 9: Table. S8). We further constructed a luciferase reporter experiment and found that luciferase activity was significantly changed with different alleles of *rs384957047* (T > C) in *DGAT1* (*t*-test: *P* < 0.01, Fig. 7D and Additional file 10: Table. S9). Overall, these observations suggest that classifying REs by evolutionary conservation can improve our understanding of the genetic mechanisms underlying complex traits and target causal variants in both livestock and humans.

## Discussion

Gene regulatory divergence and its associations with complex traits have been extensively studied in humans [50]. However, little is known about the mechanisms underlying the regulatory roles of epigenetic variation in gene expression patterns and phenotypic divergence during ruminant evolution. Here, we provide a comprehensive insight into the comparative epigenomics of the liver across three ruminants (cattle, sheep, and goats) and three non-ruminants (pigs, humans, and mice). By integrating histone signals (HK4me3 and H3K27ac), transcriptomes, chromatin accessibility, and DNA methylation, we found that genes linked to RS-REs were significantly enriched in immune processes, including immune effector processes and positive regulation of the immune system processes. Moreover, we found specific enrichment of IRF motif families in RS-REs. The liver plays an essential role in many immune processes. For example, hepatocytes are responsible for the production of 80%–90% of the circulating innate immunity proteins in the body, and the liver contains a large number of resident immune cells. Therefore, we inferred that RS-REs may be important for the ruminant inflammatory process. However, further functional experiments are required to validate the immune functions of these RS-REs [51, 52]. By combining with large-scale GWAS summary datasets for humans and cattle, we further demonstrated the relationships between complex traits and conserved/lineage-specific REs, and provided an important resource for narrowing down the range of causative mutations. Collectively, our results provide insight into the ruminant regulatory landscape and evolution. Our study also demonstrates that the datasets used for this study from public databases provide valuable functional information for the genetic breeding of livestock. Moreover, we found that 70%–90% and 9%–45% of the currently detected promoters and enhancers in humans and mice overlapped with those previously reported (Additional file 1: Fig. S12) [17, 53, 54], implying a high concordance between the current study and previous reports, especially for promoters. However, further functional validation of those enhancers required experimental validations (e.g., enhancer reporter vectors [55]) either in vivo or in vitro.

Cross-species epigenomic comparison has been widely used to investigate the evolutionary basis of REs and their impacts on species-specific complex traits (e.g., cognitive traits in humans) [11, 56]. Fang et al. observed that conserved hypomethylated regions between humans and cattle play an important role in immune response and are significantly enriched for GWAS signals of immune-related traits in both species [56], indicating that cross-species comparison of epigenome could contribute to narrowing down genetic variants of complex traits and diseases. Castelijns et al. demonstrated that human-specific REs were involved in oligodendrocyte function postnatally and were changed in the brain of autism patients, by comparing the H3K27ac mark in the brain across humans, chimpanzee, marmoset, macaque, and mice [11]. Here, through integrating with large-scale eQTLs and GWAS in both humans and cattle, we demonstrated how cross-species comparison of functional genomes can help us understand the evolutionary and genetic basis of complex traits/diseases. Overall, these observations suggest the importance of using cross-species datasets to decipher the mechanisms of GWAS loci.

Our integrative analysis of histone modification marks, DNA methylation, gene expression, and chromatin accessibility shed light on the regulatory patterns of co-evolution during ruminant evolution. We observed that the phylogenetic relationships of species based on epigenomic signal intensity and gene expression levels were consistent with that inferred from the DNA sequence, reflecting a co-evolution pattern of the functional genome and DNA sequence across species. Previous studies have proposed the notion that changes in regulatory elements affect gene expression levels and then lead to phenotypic diversity across species [36]. Zhou et al. reported that 40% of the variance in gene expression of lymphoblastoid cell lines could be explained jointly by evolutionary changes of five marks (i.e., H3K4me1, H3K4me3, H3K27ac, and H3K27me3, and RNA polymerase II) across primates [57]. These observations imply a synergistic effect of epigenomic marks on gene expression during mammalian evolution. However, such interaction effects among different types of epigenomic marks need to be further explored. We observed higher conservation of gene expression than regulatory marks. This may due to (1) REs found in this study could still contain false positives and there are still missing REs, (2) RNA-seq only measures gene expression changes at the gene level but not transcript level, as REs changes might be contributing to more varied transcript-level expression changes. Overall, a better understanding of buffering mechanisms underlying gene expression might help us interpret how evolutionary changes in epigenetic regulation contribute to gene expression variation across species.

In this study, we observed significant enrichment of GWAS signals in AC-REs across six mammals, which is consistent with the findings of Liu et al. [13]. For instance, AC-Enhancers were significantly enriched for immune-related traits in both cattle (i.e., SCS) and humans (i.e., primary biliary cirrhosis). Notably, the majority of complex traits were enriched in AC-REs in humans, especially for metabolic diseases, such as nonalcoholic fatty liver disease and alanine aminotransferase. The strong association between metabolic diseases and AC-REs suggests that important metabolism-related mutations may exist in ancient REs and also imply that cattle may serve as a biomedical model for studying human metabolic diseases.

A previous study on humans also found that human-specific regulatory gains were significantly enriched for REs depleted in autism spectrum disorder and were associated with susceptibility to neural diseases [11]. We also found that CS-REs were significantly enriched for GWAS signals of many complex traits in cattle. This may be because that most of these analyzed phenotypes (e.g., milk, protein, and fat yield) in cattle are economic traits that are under intensive artificial selection [58], while most analyzed phenotypes in humans are complex diseases that are more likely to be under natural selection [59]. Moreover, all the GWAS traits being analyzed here are somehow related to milk production in Holsten dairy cattle. The enrichment of GWAS signals of cattle traits in CS-REs may also imply the importance of studying cattle functional genomes to enhance their genetic improvement. In particular, we identified a potential causative variant for the protein percentage of milk, which is located in CS-Enhancer. It might influence the protein percentage of milk might by regulating the expression of *DGAT1* (the upstream of 1444bp away). The liver plays an important role in the synthesis and secretion of lipoproteins to provide the mammary gland with cholesterol and triglycerides for milk production during lactation in cows. Previous studies also observed that inhibition of *DGAT1* expression in bovine hepatocytes reduced triacylglycerol accumulation and significant effects of *DGAT1* on milk production traits [60, 61]. Moreover, the expression of *DGAT1* in the liver was significantly associated with milk production according to transcriptome-wide association studies in cattle [9, 62]. We found that the allele T of rs384957047 increased the expression level and transcriptional activity of *DGAT1* compared with allele C, which implied that the allele T of rs384957047 was associated with increased milk production. However, further analysis is needed to determine the functional impact of rs384957047 on milk production traits. Of note, the limitation of the current study is that only three ruminants were examined. In the future, other Bovidae species (e.g., yak and water buffalo) will be required to further explore whether this enhancer of *DGAT1* only exists in cattle. Overall, the link between cattle functional genome evolution and complex traits may reflect cattle-specific transcriptional programs and provide priority candidate regions for identifying causative variants of complex traits.

## Conclusions

Collectively, our study systematically annotated and compared regulatory elements in the liver across three economically important ruminants (i.e., cattle, sheep, and goats) and three non-ruminant mammals (i.e., pigs, mice, and humans). By integrating large-scale GWAS summary datasets in cattle and humans, we demonstrated the importance of cross-species comparison of REs in understanding the evolution of the functional genome, and genetic mechanisms underlying complex traits/diseases in livestock and humans.

## Methods

### Animal and tissue collection

In this study, a total of nine liver samples were collected from adult healthy individuals, including three cattle (3–4 years old; Holstein, healthy), three sheep (2–3 years old; Texel, healthy), and three goats (2–3 years old; Yunnan black goat, healthy). All samples were collected immediately postmortem and stored in liquid nitrogen. All animal procedures were performed according to protocols of the Institutional Animal Care and Use Committee (IACUC) at China Agricultural University. The ChIP-seq, WGBS, and RNA-seq liver assays were constructed from the same samples. For pig, mouse, and human data, only samples from adult and healthy individuals were considered.

### ChIP-seq datasets

We conducted ChIP according to published protocols [63]. The frozen liver samples were first powdered and washed with phosphate-buffered saline (PBS) buffer solution, and it was then vigorously shaken with 1% formaldehyde solution for 20 min at room temperature to cross-linking. Next, the samples were incubated at room temperature for 10 min by adding 250 mM glycine. The cross-linked liver samples were homogenized in a Dounce homogenizer and rinsed twice with PBS to solubilize DNA-protein complexes. The chromatin was sheared to an average size of 300 bp with sonication and centrifuged at 4 °C for 10 min to remove the pellet. Chromatin extracts were used to perform ChIP experiments using antibodies against H3K27ac (Abcam ab4729) and H3K4me3 (Abcam ab85850). We also constructed control experiments (input libraries) to assess potential artifacts related to the shearing of DNA and amplification, where DNA has been cross-linked and sonicated chromatin without immunoprecipitation. ChIP sequencing libraries were prepared from input or ChIP-input DNA in 96-well plates and sequenced on an Illumina HiSeq 2500 with paired-end 150 bp reads by Novogene (Beijing, China).

The public liver ChIP-seq (H3K27ac and H3K4me3) datasets were obtained from the National Center for Biotechnology Sequence Read Archive (NCBI SRA) database, including human (PRJEB6906) [17], mouse (PRJEB28147), and pig (PRJEB28147) datasets [18].

### ChIP-seq data mapping and quality evaluation

We used Trim Galore v0.4.0 and FastQC v0.11.2 software to trim the raw reads and obtain sequence quality reports. The clean reads were mapped to the corresponding reference genome of each species using BWA-MEM software v0.7.17 [64] with the default parameters: ARS-UCD1.2, Oar_rambouillet_v1.0, ARS1, Sscrofa11.1, GRCh38, GRCm39. Mapped reads were further filtered using SAMtools view utilities (1.10) [65] with the parameters “-q 1.” Duplicated alignment reads were removed using the Picard Tools v2.25.0 (https://github.com/broadinstitute/picard) with the parameter “REMOVE_DUPLICATES = true.” To evaluate the enrichment efficiency of ChIP-seq, we examined all libraries of the relative strand cross-correlation coefficient (RSC) and normalized strand cross-correlation coefficient (NSC) with phantompeakqualtools v1.2.2 [66], where an RSC >1 and NSC >1.1 indicate that the effect of antibody enrichment was acceptable in ChIP-seq. Detailed ChIP-seq quality statistics are shown in Additional file 2: Table S1A.

### ChIP-seq signal saturation and peak calling

To exclude the influence of sequencing depth and ensure that the ChIP-seq signal was saturated in all libraries, we subsampled mapped the reads from each library (H3K27ac, H3K4me3, and input libraries), starting with 5 million reads and a step of 2.5million. For the single-end sequencing data of public ChIP-seq data, we have a maximum subsample of 30 million reads. We subsampled a maximum of 40 million reads from our paired-end ChIP-seq datasets. For each subsample, we identified narrow peaks using MACS2 v2.1.1 [67] with options “-q 0.01.” Reproducible peaks were detected in at least two biological replicates and at least 50% overlap of their length using intersectBed from bedtools v2.30.0 [68]. Consensus peaks were obtained by merging all reproducible peaks of all biological replicates for further analysis. The sequencing results revealed that H3K27ac and H3K4me3 reached saturation of consistent peak detection at 20 million reads in the single-end ChIP-seq, while H3K27ac and H3K4me3 reached saturation at 37.5 million reads in the paired-end ChIP-seq (Additional file 1: Fig. S2). Subsequently, we used the subsample of H3K27ac and H3K4me3 with 20 million reads in the single-end ChIP-seq and with 37.5 million reads in the paired-end ChIP-seq for all further analyses. For one H3K4me3 library in pigs and one input library in humans, we used all the reads instead of subsampled, as their deduplicated read counts were less than 20 million.

### Identification of regulatory regions

Within each species, the consensus peaks of H3K27ac and H3K4me3 overlapped to define regulatory regions enriched for H3K27ac only or both using intersectBed from bedtools v2.30.0. We defined two categories of regulatory regions: (1) promoter, which was simultaneously marked by H3K4me3 and H3K27ac with at least 50% overlap of their length, and (2) enhancer, which contained H3K27ac peaks and did not overlap with H3K4me3 enriched regions. Histone modifications could be used to identify putative REs (i.e., enhancers and promoters) at a genome scale. However, these REs are required for further functional validation, such as using enhancer reporter vectors either in transfected cells or transgenic animals (e.g., mice) [69, 70]. The statistics of regulatory regions are shown in Additional file 3: Table S2.

### Comparison with those previously detected

To validate the accuracy of REs mapping, we downloaded H3K27ac/H3K4me3 ChIP-seq data for the livers of cattle (PRJEB6906, PRJNA665199, PRJNA665216 and PRJEB41939) [17, 19, 20], pigs (PRJNA597497) [15], and mice (PRJEB6906) [17] from the SRA database (Additional file 11: Table S10). The REs were identified using the aforementioned methods. Mouse and human REs identified in the current study were compared to REs annotated in Ensembl version 103 [53] and VISTA [54] (downloaded July 28, 2022). The overlap of REs identified in the current study and those confirmed in the previous study was calculated using the intersect command in BEDTools v2.30.0 with parameters “-f 0.5 –F 0.5 -e.” Moreover, we obtained the chromatin states of cattle [19], pigs [19], humans [5], and mice [71] in the liver and performed enrichment analysis with the 1000 permutation test using the R package regioneR [72].

### Whole genome alignment and cross-species comparisons

Cross-species comparisons were performed using 46 eutherian mammals Enredo-Pecan-Ortheus (EPO) multiple genome alignments available from ENSEMBL Compara API (v103) [73, 74]. To further identify the highly conserved REs across the six species, we used cattle as the central species and then aligned other species to cattle in a pairwise manner. REs were considered highly conserved if they overlapped with another regulatory region in all species with a minimum length of 50%. Similarly, lineage-specific REs were defined for ruminants using the methods as those used to identify highly conserved regions. REs were compared between the reference species (cattle) and species in the other clade using EPO multiple genome alignments. Lineage-specific REs were highly conserved across species within the clade (i.e., cattle, sheep, and goats for the ruminant branch) but not in other species.

### Identification of species-specific REs

The species-specific REs were determined as previously described [18]. The REs were defined as species-specific (*N*_*si*_, see Eq. 2) if the DNA sequence could not be aligned to any other species, or if RE activity could not be detected in any other species with the underlying DNA sequence alignable across species.

1 $${N}_i={N}_{Ni}+{N}_{Li}+{N}_{Ci}$$

2 $${N}_{si}={N}_{Ni}+{N}_{Li}$$

where *N*_*i*_ = number of REs (promoters and enhancers) in the species *i* (*i*=cattle, sheep, goats, pigs, humans, and mice); *N*_*Ni*_ = number of REs in species *i* without DNA sequence alignment to any other species. REs without DNA sequence alignment were defined as REs that could not be mapped to orthologous regions using 46 eutherian mammals Enredo-Pecan-Ortheus (EPO) multiple genome alignments in ENSEMBL (v103); *N*_*Li*_ = number of REs in species *i* without sharing of regulatory activity in any other species but with DNA sequence alignment across many species; *N*_*Ci*_ = number of REs in species *i* with shared regulatory activity in at least one other species; *N*_*si*_ = number of species-specific REs in the species *i*.

### RNA sequencing library construction

Total RNA was extracted using TRIzol method from frozen liver samples of three ruminant species: cattle, sheep, and goats. mRNA was enriched using magnetic beads with Oligo (dT) and sheared. The cDNA was synthesized using random hexamers, end-repaired, and ligated to A base and sequencing adapters. Then, cDNA libraries were obtained by polymerase chain reaction (PCR) amplification and sequenced using the Illumina Hiseq X Ten platform with 150bp-paired reads (Novogene Inc., Beijing, China). The liver RNA-seq datasets were obtained from the NCBI SRA database, including PRJEB13074 for humans [36], PRJEB33381 for mice, and PRJEB33381 for pigs [18].

### RNA sequencing mapping and gene expression quantification

The adapter sequences and low-quality bases were filtered using Trimmomatic v0.39 [75]. Detailed RNA-seq quality statistics are shown in Additional file 2: Table S1B. The trimmed reads were mapped to the Ensembl 103 version of reference genomes with annotated genes using STAR aligner v 2.7.6 [76] with the following options: “--outFilterMismatchNmax 3 –outFilterType BySJout –quantMode GeneCounts –outFilterMismatchNoverLmax 0.04 –outSAMtype SAM SortedByCoordinate.” Gene expression levels were quantified as counts using featureCounts [77] and were normalized by Trimmed Mean of M value (TMM) with edgeR [78]. Genes with TMM-normalized counts per million (CPM) of more than 1 in 20% of the samples were used for further analysis.

### WGBS library construction and analysis

The genomic DNA of liver samples from three ruminants (cattle, sheep, and goats) was extracted according to the TIANamp Genomic DNA Kit protocol (TIANGEN, Beijing, China). The qualified DNA was fragmented to 300bp and subjected to terminal repairing and an addition at the 3′-terminus. Bisulfite conversion of DNA was performed using the ZYMO EZ DNA Methylation-Gold Kit. DNA fragments were amplified using PCR and selected for library fragment size. The quantified library was sequenced on the Illumina HiSeq X Ten machine (PE-150 bp FC; Annoroad, Beijing, China). The liver WGBS datasets were obtained from the NCBI SRA database as well, including PRJNA287622 for humans [79], PRJNA416505 for mice [80], and PRJNA357500 for pigs [81]. The cattle WGBS datasets were obtained from the NCBI SRA database under PRJNA612978 and PRJNA417285 [26, 82], representing five tissues, i.e., adipose, cortex, lung, muscle, and spleen.

Raw reads were trimmed using Trim Galore v0.4.0 with default parameters. The clean reads were mapped to the reference genome using bismark v0.23.0 with default parameters and were deduplicated with the deduplicate_bismark options [83]. CpGs with a coverage of at least five were used for further analysis. The methylation levels of CpG sites were calculated using MethPipe v5.0.0, according to the number of methylated Cs in reads at the position corresponding to the site divided by the total of methylated Cs and unmethylated Ts mapping to that position [84].

### Assay for transposase-accessible chromatin with high-throughput sequencing (ATAC-seq) processing

We obtained 23 public ATAC-seq datasets from SRA databases, and the accession numbers are listed in Additional file 2: Table S1D. ATAC-seq raw reads were filtered using Trim Galore v0.4.0 with the following parameters “-q 25 -length 25 -e 0.1 --stringency 4.” The clean reads were mapped to the reference genome, and PCR duplicates were eliminated using Bowtie v2.4.2 and Picard Tools v 2.25.0. ATAC-seq peaks were called using MACS2 v 2.1.1 with the following parameters “--nomodel --shift 37 -B --SPMR --ext 73 --pval 1e-2 --call-summits.” The replicated peaks that overlapped by at least 50% in the two samples were identified using intersectBed from bedtools v2.30.0.

### Assignment of REs to putative target genes and transcriptome divergence

Putative target genes of the REs were identified using methods similar to the GREAT v4.0.4 [85]. The regulatory association domains of genes were defined as 5kb upstream and 1kb downstream from their TSSs. The REs were then linked to their putative target genes if they overlapped (≥1bp) with regulatory association domains of genes using the intersect command in BEDTools v2.30.0. We divided genes into two groups according to the number of REs, such as single (e.g., the number of promoters≤1) vs. multiple (e.g., the number of promoters≥2). When estimating the relative conservation of genes with different numbers of REs, we considered single and multiple genes with matched expression levels using the MatchIt [86] library in R with the option “caliper=0.5.” The transcriptome evolutionary divergence was measured by Spearman’s correlation coefficients of expression levels of orthologous genes between pairs of species. Divergence time was obtained from Timetree (http://www.timetree.org/).

### Regulatory and transcriptome phylogenies

We constructed phylogenic trees based on pairwise distance matrices using the neighbor-joining method in the ape R library [87]. We obtained 9796 1:1 orthologous genes from Ensembl release 103 (http://asia.ensembl.org/info/genome/compara/homology_method.html) [88], which showed CPM > 1 in more than 20% of the samples. We then calculated gene expression levels and four marker (H3K4me3, H3K27ac, chromosomal accessibility, DNA methylation levels) signal intensities in REs (promoters and enhancers) of orthologous genes. Pairwise distance matrices were estimated as 1− *ρ* (*ρ* is Spearman’s correlation coefficient of species). The branch length of phylogenetic trees was accessed with 1000 bootstrap analyses.

### Gene function and TF motif analyses

Gene function enrichment analyses were performed for genes associated with lineage-specific REs using GREAT v4.0.4 for biological process ontologies. The regulatory domain was defined with the parameters: 5kb upstream and 1kb downstream from TSSs (extending up to 50kb in both directions for the regulatory domains of nearest genes). Significantly enriched terms were determined based on FDR< 0.05. We then performed motif enrichment analysis using HOMER v4.11 with the default motif database [89]. To scan lineage-specific peaks (RS-Enhancers and CS-Enhancers) for the PPARA motifs, we performed RSAT matrix-scan [90] with the matrix of PPARA (MA1148.1) from the JASPAR database (https://jaspar.genereg.net/) with the following parameters: Markov order:1, weight score ≥1.

### GWAS enrichment analysis based on REs

Most complex traits are polygenic, and recent studies have reported some differences in the effects of different functional regions on traits [13, 91]. To further understand the regulatory mechanism of economically important traits and diseases in livestock and humans, we obtained GWAS summary datasets for 44 complex traits of 27,214 Holstein bulls with 3,148,506 SNPs [3, 46]. For the human GWAS summary datasets, we collected GWAS summary datasets for 52 complex traits with an average SNPs of 10,846,391 and an average individual of 264,890. The details of the human GWAS summary datasets are summarized in Additional file 12: Table S11. We added 50-kb windows around REs to include the important cis-regulatory variants. We then employed a count-based marker-set test approach with the QGG R package to examine whether GWAS signals were enriched in candidate regulatory regions [92].

$${T}_{\textrm{count}}=\sum_{j=1}^cI\left({t}_i<{t}_o\right)$$

where *T*_count_ is the summary statistics for each candidate regulatory region, *c* is the number of SNPs in the given region, for instance, AC-Enhancers. *I* is an indicator function that takes value one when *t*_*i*_ < *t*_*o*_, and we choose *t*_*o*_=0.01 as the cut-off. The significance of enrichment was calculated using the hypergeometric test [93]. *P* < 1.0e−5 was set as the threshold of the significant enrichment. Detailed descriptions can be found in Fang et al. [25].

### Dual-luciferase reporter assays

Here, 293T cells were seeded in 96-well plates and transfected until the cell density reaches 50%–70%. Three recombinant plasmids were constructed, namely DGAT1_T, in which the SNP of DGAT1 was allele T; DGAT1_C, in which the SNP of DGAT1 was allele C; and the control plasmid pGL3-basic. MluI and XhoI were identified as the restriction sites of DGAT1 (Additional file 1: Fig. S13). The target fragment sequence contains 30bp upstream of rs384957047 and the sequence to the transcription start site of DGAT1, including enhancer and promoter. Six hours after transfection of 293T cells, fresh medium was replaced, and 48 h after transfection, samples were collected to detect the luciferase activities using a microplate system. The transfection test was repeated at least three times, and each germplasm plasmid was equipped with two replicates.

### Other downstream bioinformatics analyses

The chromatin states of four mammal (cattle, pigs, humans, and mice), were obtained from previous studies [5, 19, 71]. Enrichment analysis of chromatin state was performed using the regioneR library in R with 1000 times permutation tests. We conducted genomic variants (i.e., SNP, CNVR, QTL, and eQTL) enrichment analyses using regioneR library in R (Permutation test: 1000). The genomic variants were downloaded from public databases, including NCBI dbSNP, cattle QTLdb (Release 45, August 23, 2021), AOD [39], and a previous study [9]. We calculated the histone and ATAC signal intensities using deepTools v3.1.1 with the following parameters “--normalizeUsing CPM.”

## Supplementary Information

**Additional file 1: Figure S1.** The overview design of this study. **Figure S2.** General characteristics of ChIP-seq, ATAC-seq, and DNA methylation data in six mammals. **Figure S3.** The assessment of the mapping accuracy for putative regulatory elements (REs). **Figure S4.** Cluster analysis of the six mammals across multi-omics. **Figure S5.** Dynamic changes of REs across species. **Figure S6.** Distributions of averaged epigenomic mark signals of four different types of regulatory elements (REs) across eight tissues in cattle. **Figure S7.** The expression levels (transcripts per million, TPM) of putative target genes of six types of lineage-specific regulatory elements across cattle tissues. **Figure S8.** Phylogenetic trees of four categories of genes. **Figure S9.** The number of enhancers contributes to increasing gene expression levels. **Figure S10.** The number of promoters contributes to increasing gene expression levels. **Figure S11.** SNPs were enriched in regulatory elements (REs). **Figure S12.** Comparison of human and mouse regulatory regions in the current study with those annotated in Ensembl and VISTA. **Figure S13.** The diagram of luciferase reporters used in this study. The red boxes represent the restriction sites of *DGAT1*.

**Additional file 2: Table S1A.** Sequencing and mapping statistics of ChIP-seq, **Table S1B.** Sequencing and mapping statistics of RNA-seq, **Table S1C.** Sequencing and mapping statistics of WGBS, **Table S1D.** Sequencing and mapping statistics of ATAC-seq. **Table S1E.** The summary of human GWAS datasets.

**Additional file 3: Table S2.** Numbers and definitions of regulatory regions.

**Additional file 4: Table S3.** Significant gene ontology (GO) terms (BinomFdrQ < 1.0e-4) for six categories of lineage-specific REs.

**Additional file 5: Table S4.** Motif enrichment analysis for six categories of lineage-specific REs.

**Additional file 6: Table S5.** The summary of QTL enrichment folds in cattle.

**Additional file 7: Table S6.** The summary of significant GWAS signal enrichment in cattle.

**Additional file 8: Table S7.** The summary of significant GWAS signal enrichment in humans.

**Additional file 9: Table S8.** Transcription factor binding prediction analysis for rs384957047 mutation in the upstream region of *DGAT1*.

**Additional file 10: Table S9.** The luciferase activities with a different allele of rs384957047 (T > C) in *DGAT1*.

**Additional file 11: Table S10.** The public ChIP-seq data from the liver of cattle, pig and mouse to validate regulatory regions in the current study.

**Additional file 12: Table S11.** The summary of human GWAS datasets.

## Data Availability

All data generated or analyzed during this study are included in this published article, its supplementary information files and publicly available repositories. All raw and processed sequencing data for three ruminant liver samples generated in this study have been submitted to the NCBI Gene Expression Omnibus (GEO) database under accession numbers GSE206184 [94], GSE206511 [95], and GSE206736 [96]. The public ChIP-seq, RNA-seq, WGBS, and ATAC-seq are downloaded from SRA databases, and the accession numbers can be found in Additional file 2: Table S1 and Additional file 11: Table S10. The chromatin states of cattle, pig, mouse, and human liver are publicly available at http://farm.cse.ucdavis.edu/~ckern/Nature_Communications_2020/, https://egg2.wustl.edu/roadmap/web_portal/ and https://www.encodeproject.org/search/?searchTerm=ChromHMM+Zhiping+Weng. Cattle QTLs are download from cattle QTL database (https://www.animalgenome.org/cgi-bin/QTLdb/BT/index, Release 45, August 23, 2021). Cattle CNVRs and phyloP sequence conservation score (phyloP100way.cattle.score) are found in http://222.90.83.22:88/code/index.php/main. The pLI score is publicly available at http://hgdownload.soe.ucsc.edu/gbdb/hg19/gnomAD/pLI/. Cattle liver eQTLs can be available at https://cgtex.roslin.ed.ac.uk/. The cattle gene atlas is publicly available at http://cattlegeneatlas.roslin.ed.ac.uk. The GWAS summary statistics for cattle complex traits are publically available through Figshare (https://figshare.com/s/ea726fa95a5bac158ac1; https://figshare.com/s/94540148512dddf7ed32). Details of human GWAS summary statistics are summarized in Additional file 12: Table S11. The scripts for enrichment analysis are available at the zenodo website (10.5281/zenodo.7275349) [97]. REs annotated in this study have been deposited in the figshare database (10.6084/m9.figshare.20402178.v1 ) [98].

## Abbreviations

ChIP-seq: Chromatin immunoprecipitation followed by sequencing
RNA-seq: RNA sequencing
ATAC-seq: Assay for transposase-accessible chromatin with high-throughput sequencing
RE: Regulatory element
eQTL: Expression quantitative trait locus
WGBS: Whole genome bisulfite sequencing
GWAS: Genome-wide association studies
CNVR: Copy number variable region
SNP: Single-nucleotide polymorphism
FAANG: Functional Annotation of Animal Genomes consortium
FarmGTEx: Farm animal Genotype-Tissue Expression project
TSS: Transcription start sites
GO: Gene Ontology
TF: Transcriptional factor
TPM: Transcripts per kilobase million
EPO: Enredo-Pecan-Ortheus
LDSC: Stratified linkage disequilibrium score regression
LD: Linkage disequilibrium
MAF: Minor allele frequency
SCS: Somatic cell score
RSC: Relative strand cross-correlation coefficient
NSC: Normalized strand cross-correlation coefficient
CPM: Counts per million
