# Supplementary material for: Comparative epigenomics reveals the impact of ruminant-specific regulatory elements on complex traits
Source: BMC Biol. 2022 Dec 8;20:273. doi: 10.1186/s12915-022-01459-0 (PMC9730597; doi:10.1186/s12915-022-01459-0)
Supplement: Supplementary file 1 — Additional file 1: Figure S1. The overview design of this study. Figure S2. General characteristics of ChIP-seq, ATAC-seq, and DNA methylation data in six mammals. Figure S3. The assessment of the mapping accuracy for putative regulatory elements (REs). Figure S4. Cluster analysis of the six mammals across multi-omics. Figure S5. Dynamic changes of REs across species. Figure S6. Distributions of averaged epigenomic mark signals of four different types of regulatory elements (REs) across eight tissues in cattle. Figure S7. The expression levels (transcripts per million, TPM) of putative target genes of six types of lineage-specific regulatory elements across cattle tissues. Figure S8. Phylogenetic trees of four categories of genes. Figure S9. The number of enhancers contributes to increasing gene expression levels. Figure S10. The number of promoters contributes to increasing gene expression levels. Figure S11. SNPs were enriched in regulatory elements (REs). Figure S12. Comparison of human and mouse regulatory regions in the current study with those annotated in Ensembl and VISTA. Figure S13. The diagram of luciferase reporters used in this study. The red boxes represent the restriction sites of DGAT1. [file 12915_2022_1459_MOESM1_ESM.docx]

**
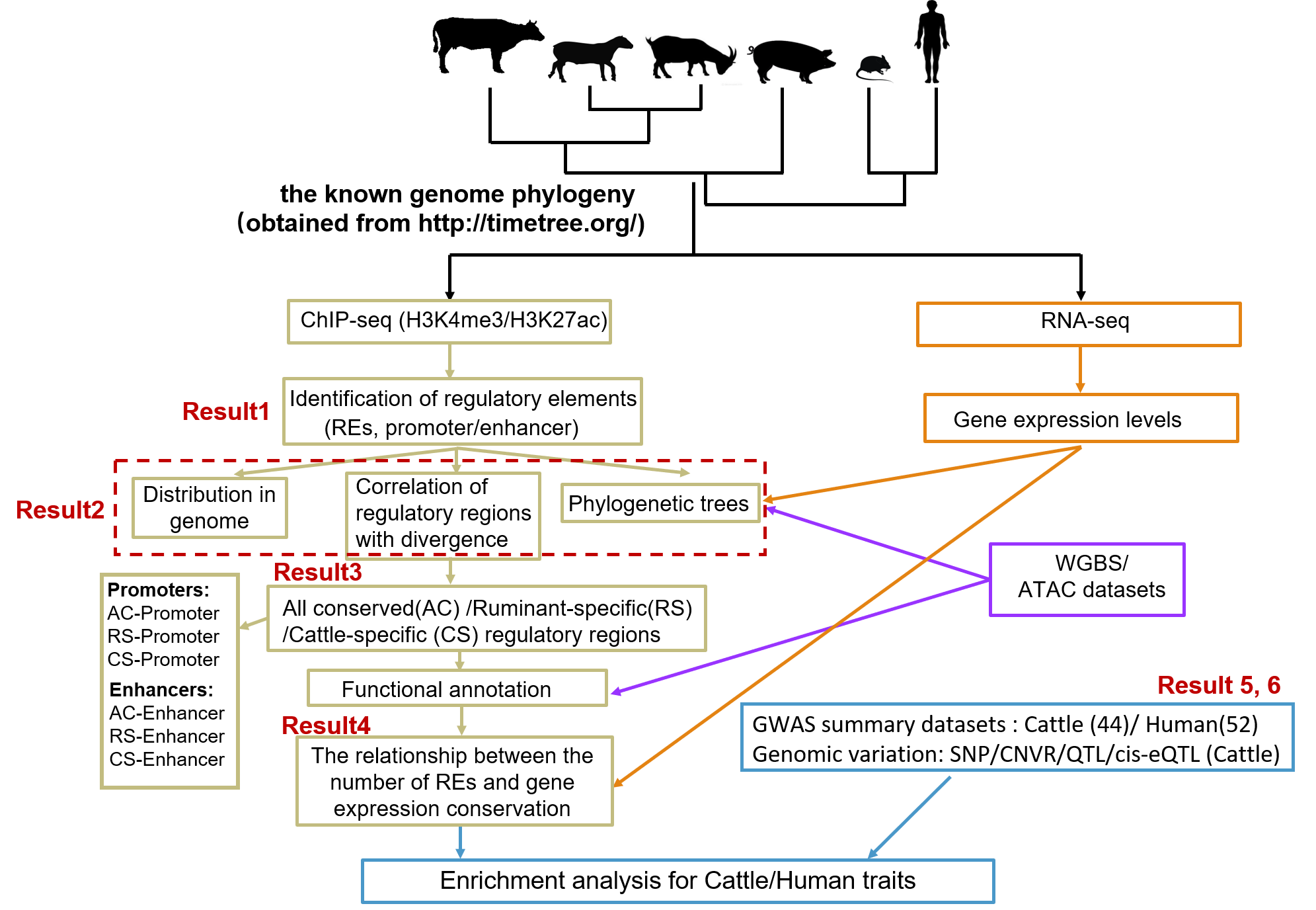
**

**Figure S1. The overview design of this study.** Using 40 chromatin immunoprecipitation sequencing (ChIP-seq) data of two histone modifications (i.e., H3K4me3 and H3K27ac) in the liver, we systematically compared the landscape of regulatory elements across six mammalian species, including cattle (n=3 individuals), sheep (n=3 individuals), goats (n=3 individuals), pigs (n=3 individuals), mice (n=4 individuals), and humans (n=4 individuals). We studied the evolutionary rates of epigenomic marks and gene expression across species. We also investigated the impact of epigenomic evolution on gene expression and complex phenotypes by integrating lineage-specific regulatory elements with expression quantitative traits loci (eQTLs) and results of large-scale genome-wide association studies (GWAS) from 44 and 52 complex traits in cattle and humans, respectively.


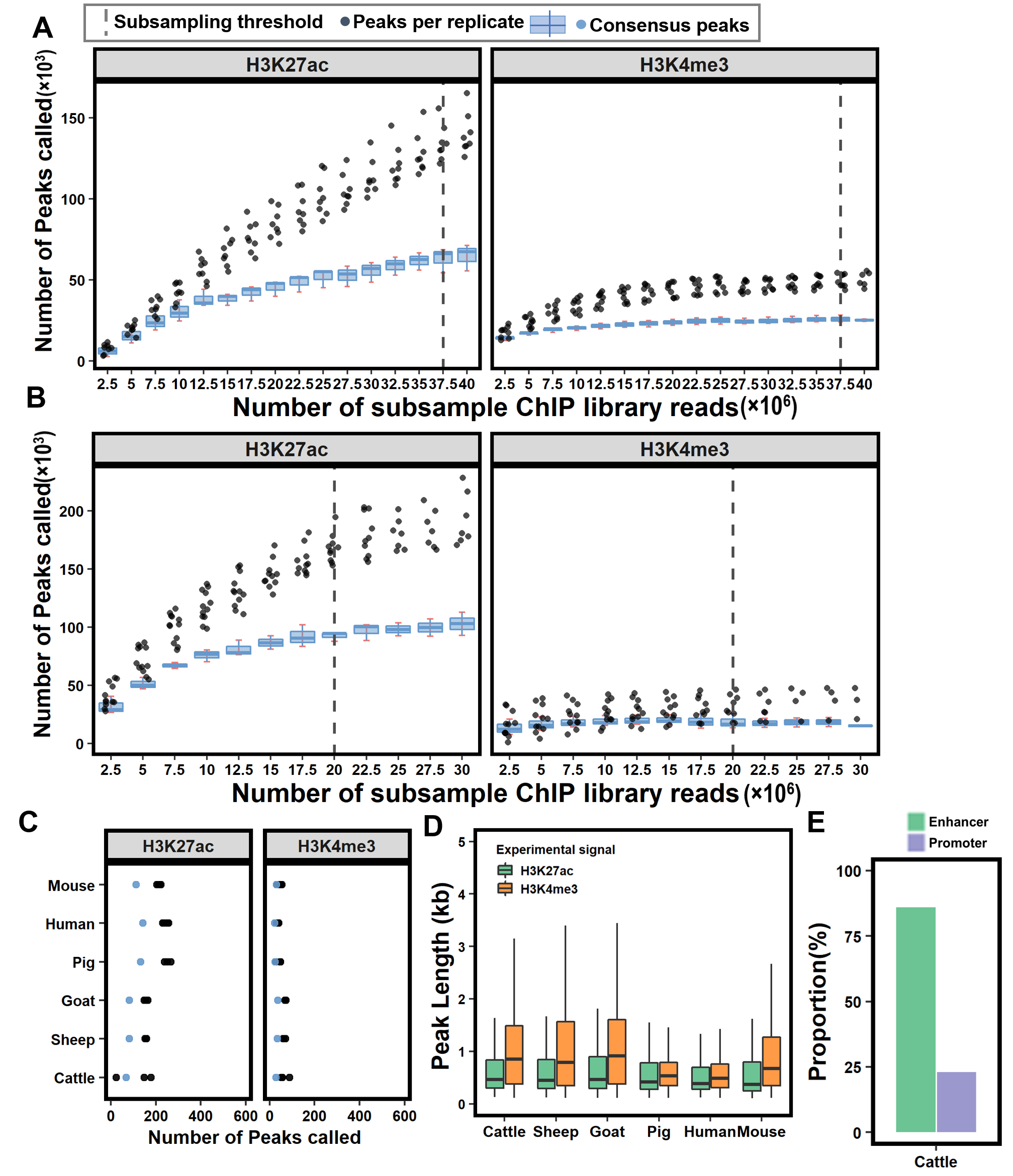


**Figure S2. General characteristics of ChIP-seq, ATAC-seq, and DNA methylation data in six mammals.** (**A** and **B**) The saturation analysis of ChIP-Seq signals for three ruminants (paired reads) and three non-ruminants (single reads). We randomly subsampled mapped reads from all 40 samples (H3K27ac and H3K4me3). We then obtained the number of consensus peaks (appear in at least two biological replicates) for each histone mark. Grey dots represent peaks per sample. Blue dots represent consensus peaks detected in at least two biological replicates. The dashed line represents the subsampling threshold of the ChIP-seq signal reached saturation. **C** Number of peaks identified for H3K27ac and H3K4me3 in each species’ liver using samples subsampled at the appropriate threshold. Black dots represent peaks per sample called. Blue dots represent consensus peaks detected in at least two biological replicates. **D** Length distribution of consensus H3K27ac (green) and H3K4me3 (orange) peaks across species. **F** Tissue-specificity of liver REs through comparing to the other seven cattle tissues-adipose, cortex, cerebellum, hypothalamus, lung, spleen, and muscle.


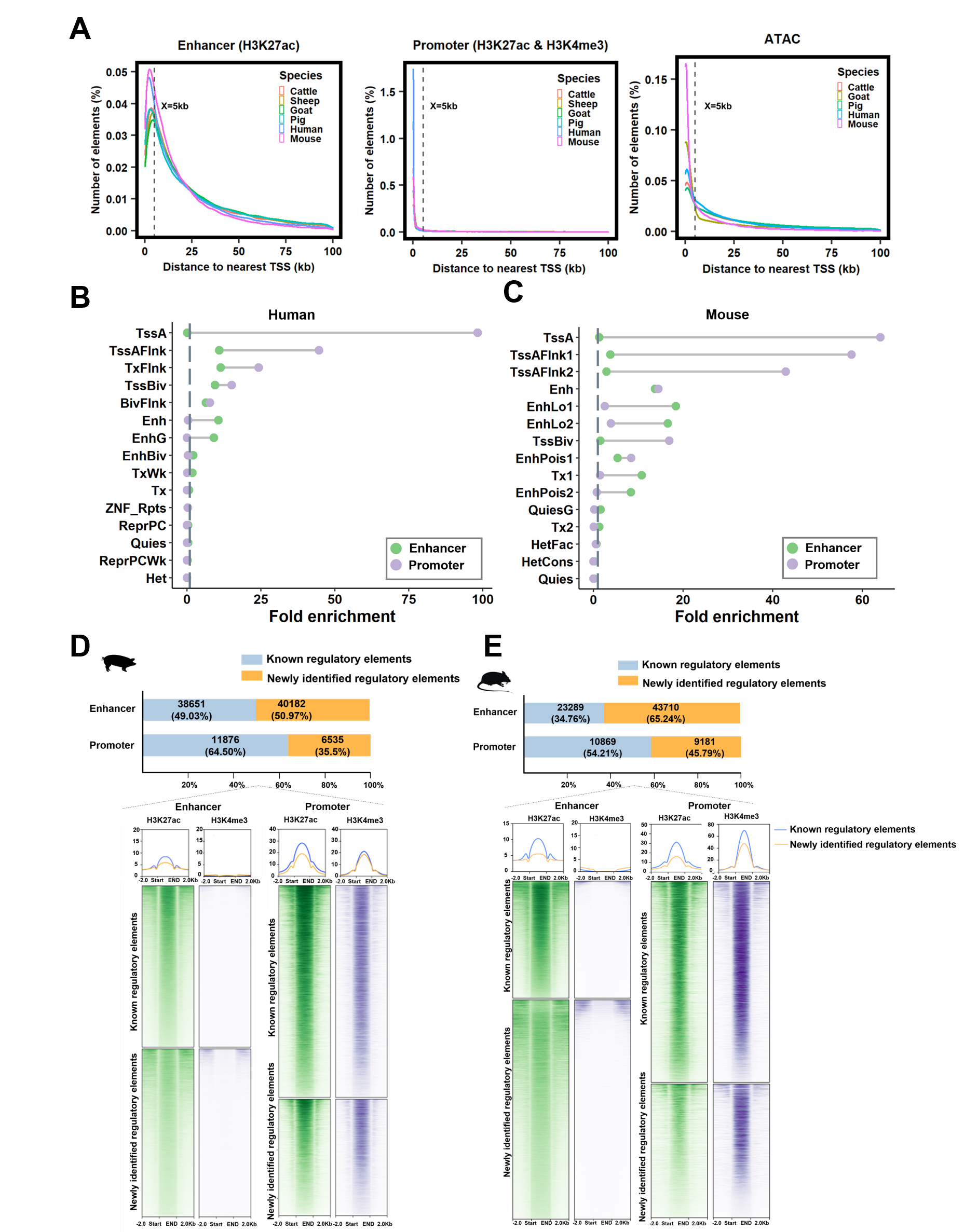


**Figure S3. The assessment of the mapping accuracy for putative regulatory elements (REs).** **A** The distribution of distances from regulatory elements to the nearest transcription start sites (TSS) in each species. (**B and C**) Distribution frequencies of regulatory elements in the 15-state ChromHMM model from human (**B**) and mouse (**C**) liver tissue. (**D and E**) Percentages of regulatory regions newly detected in the current study (orange) and recovered by published data (blue) from pig (**D**) and mouse (**E**) liver tissue.

**
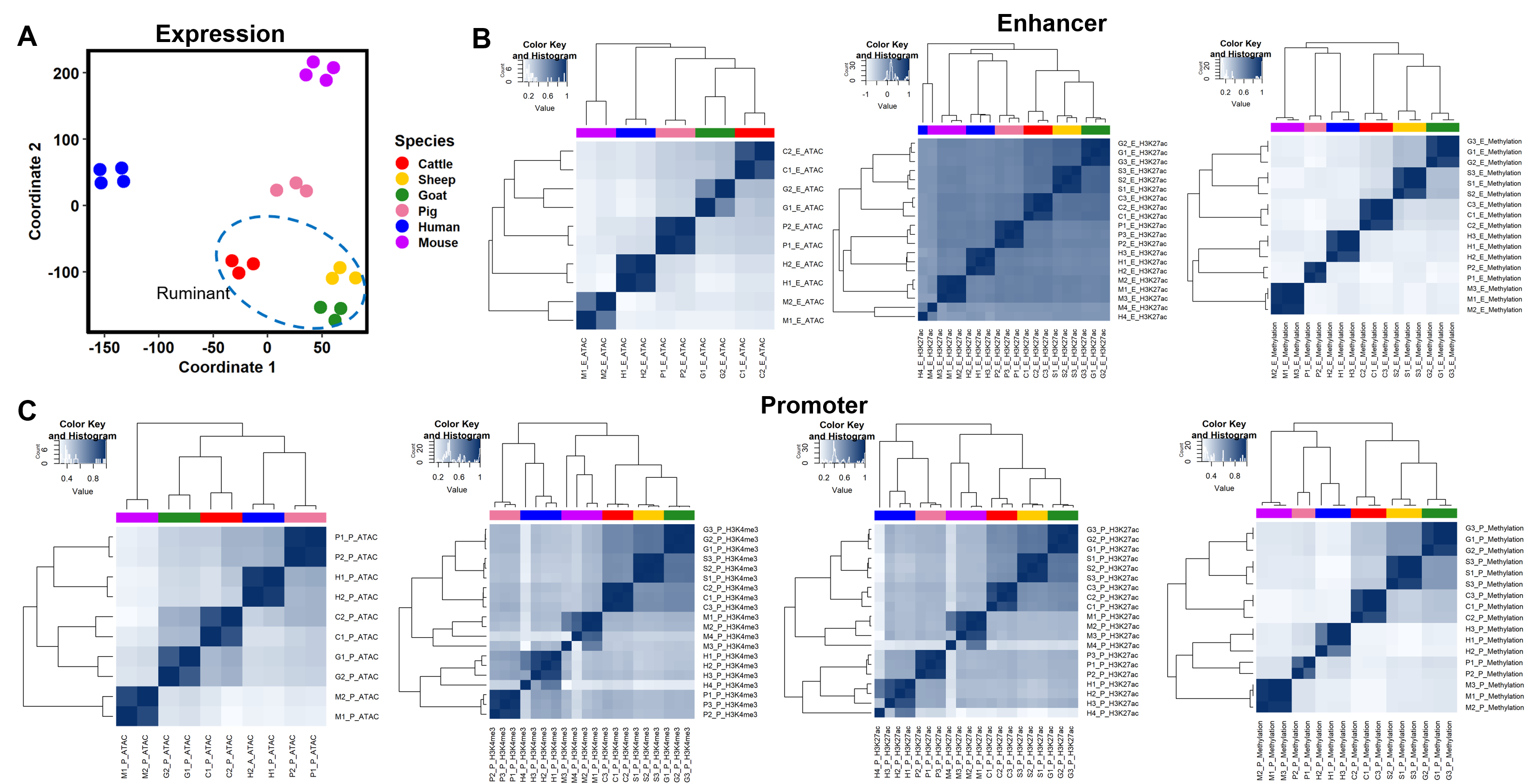
**

**Figure S4. Cluster analysis of the six mammals across multi-omics.** **A** t-Distributed Stochastic Neighbor Embedding analysis (t-SNE) of gene expression level across six mammals. (**B and C**) Heatmap based on ATAC-seq signal intensities, histone modification signal (H3K27ac and H3K4me3), and DNA methylation level in the enhancers (**B**) or promoters (**C**) of orthologous genes.


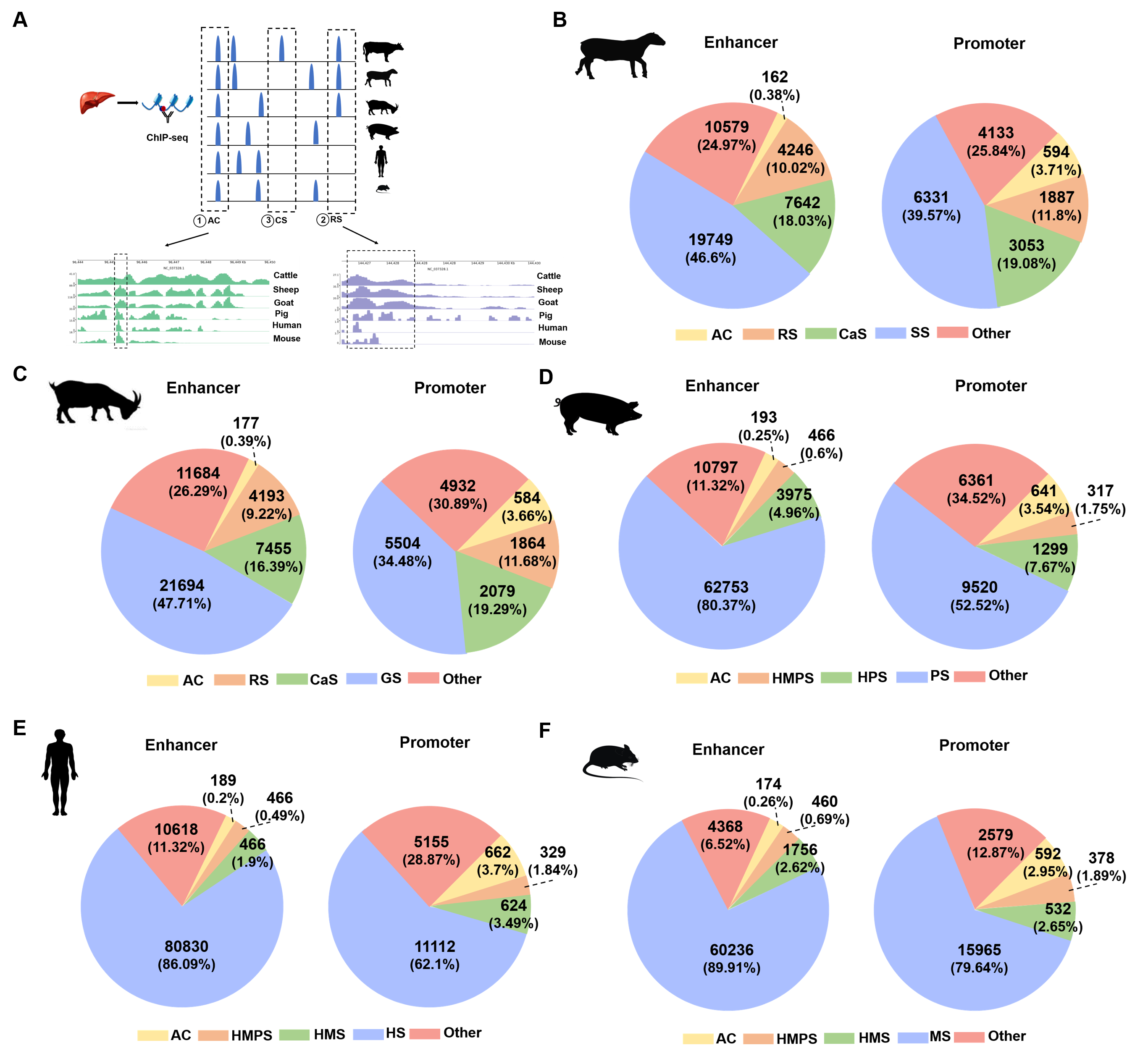


**Figure S5.** **Dynamic changes of REs across species.** **A** The identification for six types of REs in cattle. (**B-F**) The fractions of lineage-specific REs only on the chromosomes in sheep (**B**), goat (**C**), pig (**D**), human (**E**) and mouse (**F**).


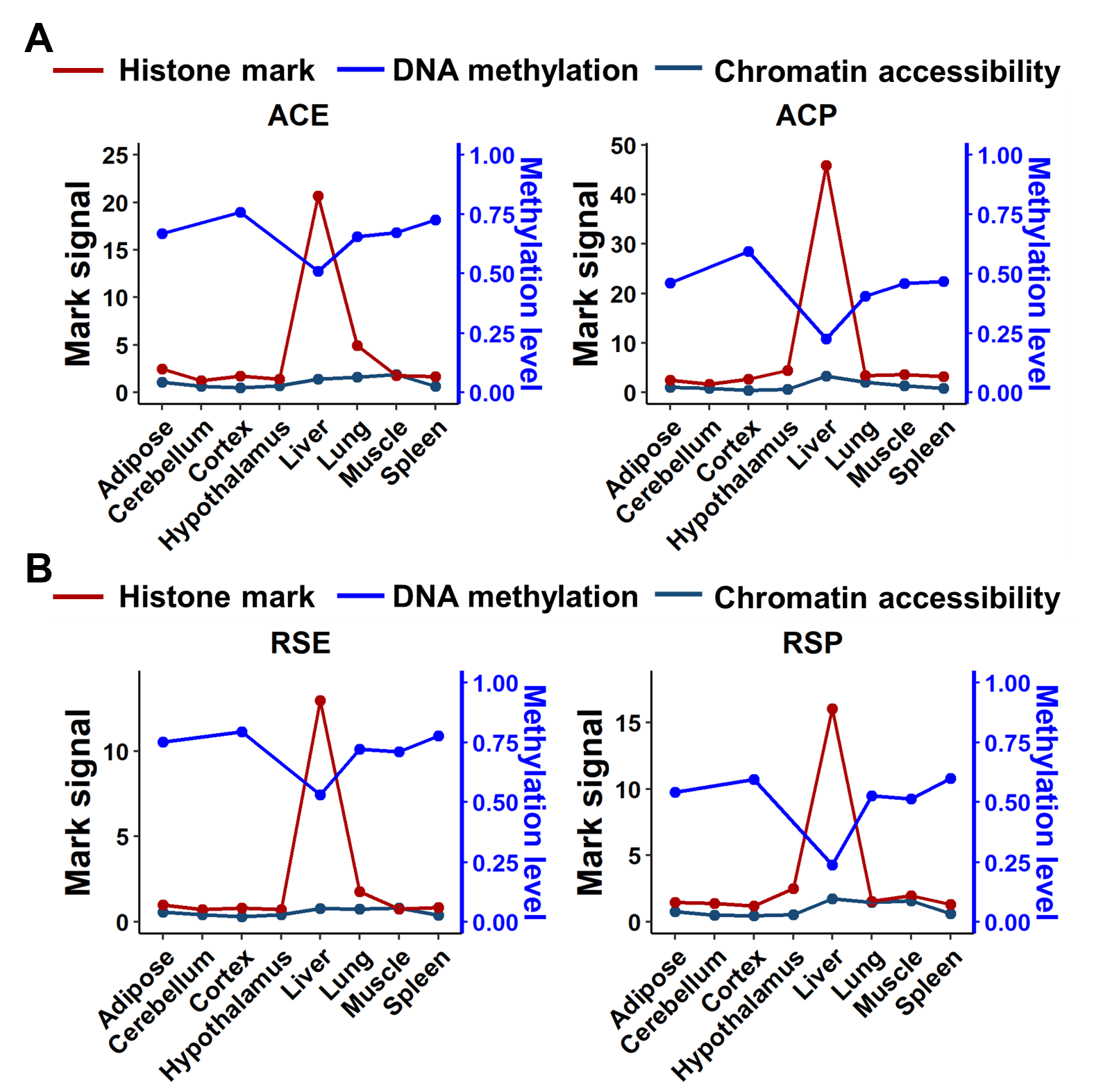


**Figure S6. Distributions of averaged epigenomic mark signals of four different types of regulatory elements (REs) across eight tissues in cattle.** Epigenomic marks include H3K4me3, H3K27ac, DNA methylation, and chromatin accessibility. The four types of REs are highly-conserved promoters (ACP), ruminant-specific promoters (RSP), highly-conserved enhancers (ACE), and ruminant-specific enhancers (RSE).


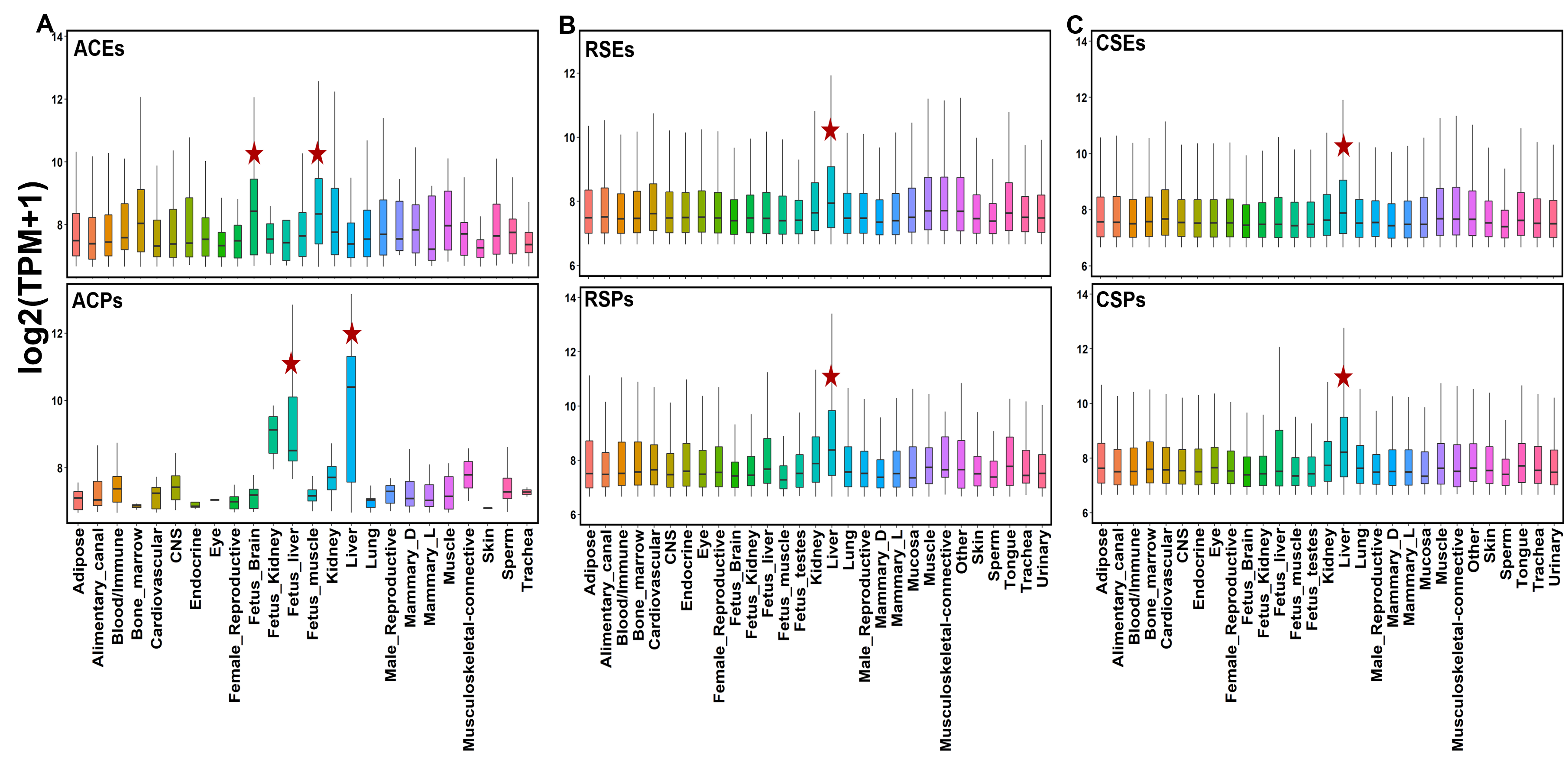


**Figure S7.** The expression levels (transcripts per million, TPM) of putative target genes of six types of lineage-specific regulatory elements across cattle tissues. The expression data were downloaded from the cattle gene expression atlas.


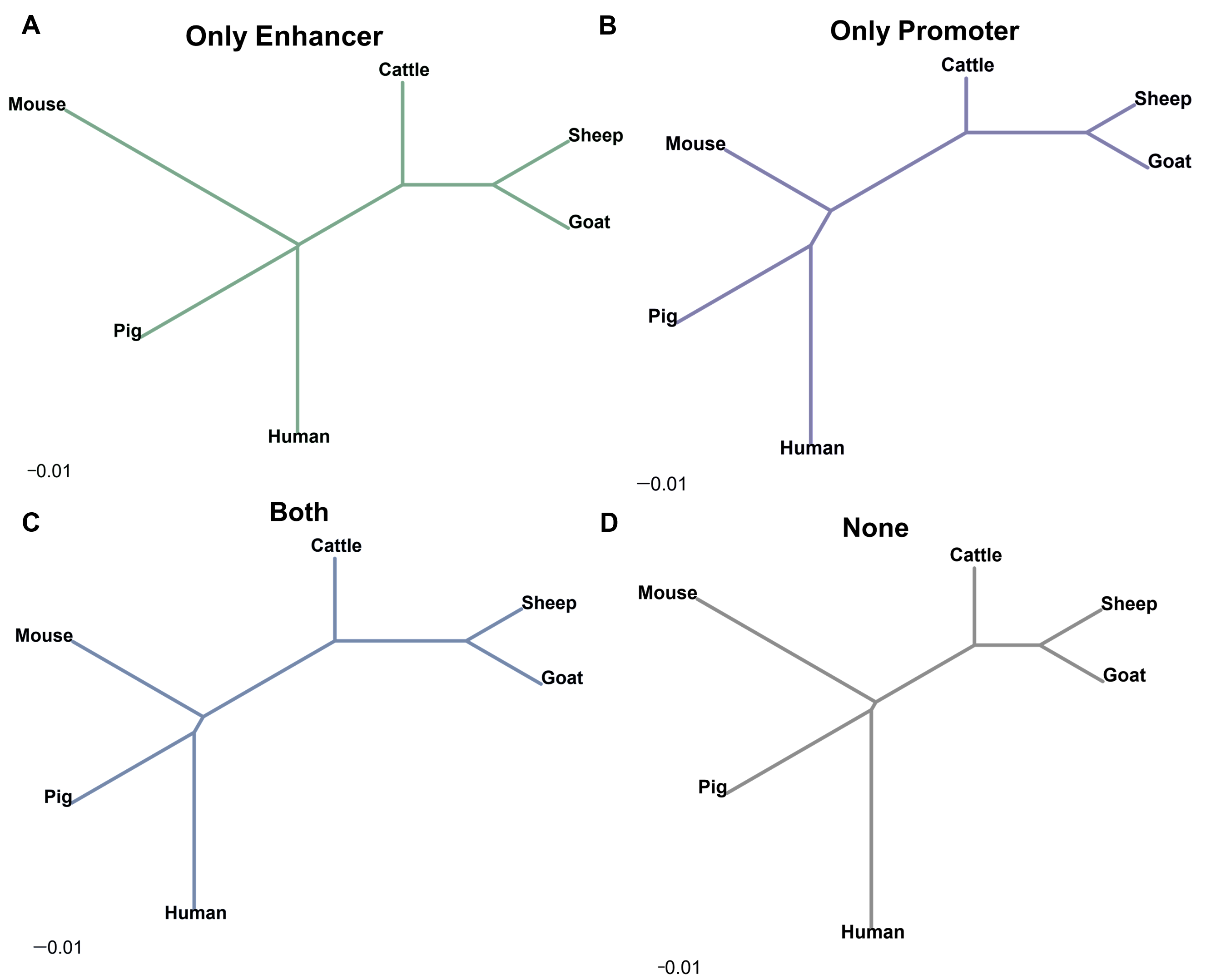


**Figure S8. Evolution of four categories genes.** The phylogenetic trees using neighbor-joining (NJ) method for genes associated with only enhancers (A), only promoters (B), both enhancers and enhancer (C), and none REs (D), respectively.


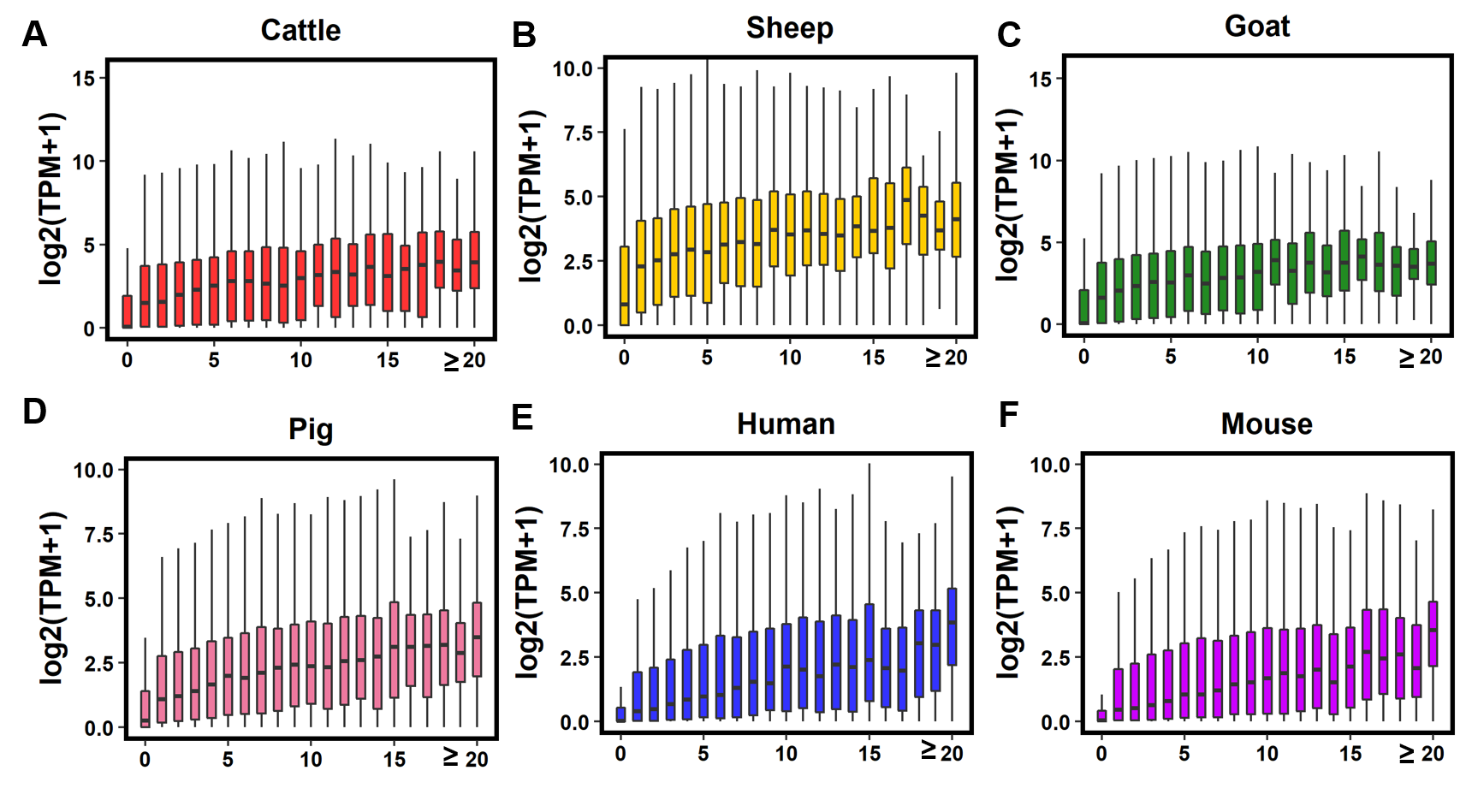


**Figure S9. The number of enhancers contributes to increasing gene expression levels.** Box plots shows the dynamic changes of gene expression level with increasing numbers of enhancers across six mammals, including cattle (A), sheep (B), goat (C), pig (D), human (E), and mouse (F).


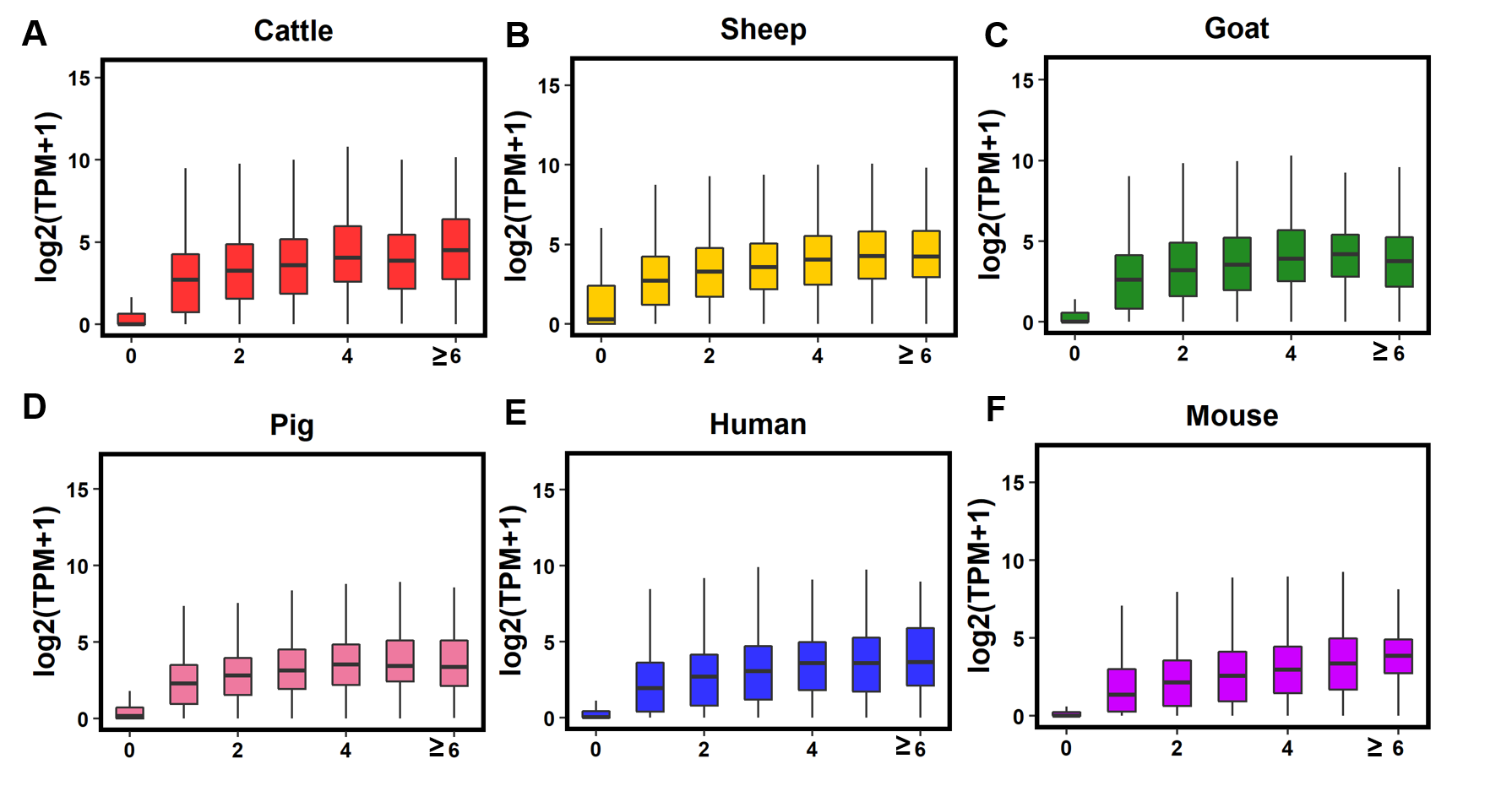


**Figure S10. The number of promoters contributes to increasing gene expression levels.** Box plots shows the dynamic changes of gene expression level with increasing numbers of promoters across six mammals, including cattle (A), sheep (B), goat (C), pig (D), human (E), and mouse (F).


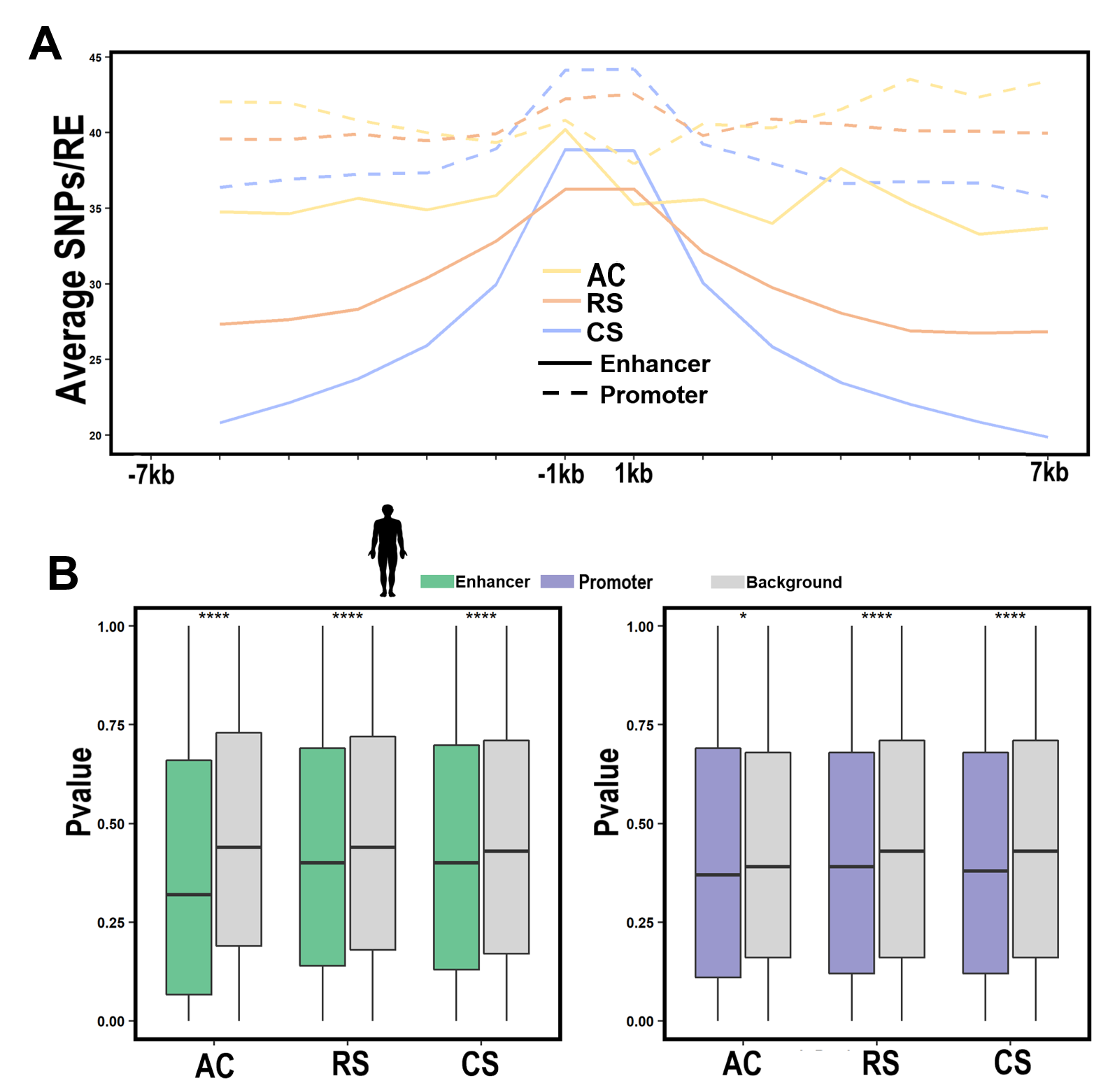


**Figure S11. SNPs were enriched in regulatory elements (REs).** A The number of SNPs flanking the REs for cattle. B The P-values of SNPs inside and outside of REs from GWAS summary datasets for alkaline phosphatase in humans.


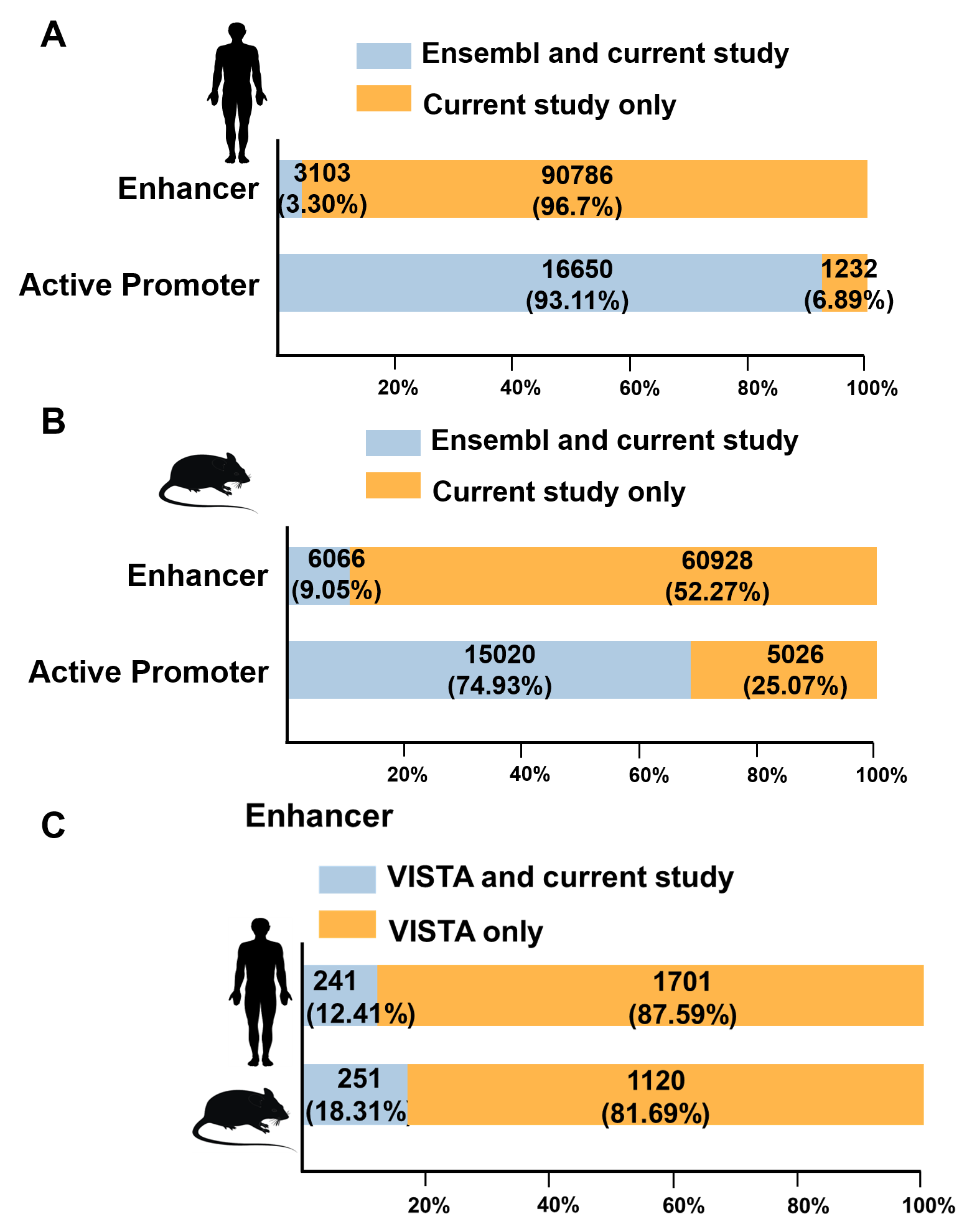


**Figure S12. Comparison of human and mouse regulatory regions in the current study with those annotated in Ensembl and VISTA.** (**A and B**) Percentages of regulatory regions newly detected in the current study (orange) and recovered by Ensembl version 103 (blue) from human hepatocyte (**A**) and mouse liver tissue (**B**). **C.** Percentages of regulatory regions detected in VISTA database (orange) and recovered by the current study (blue) from human and mouse (downloaded July 28, 2022).


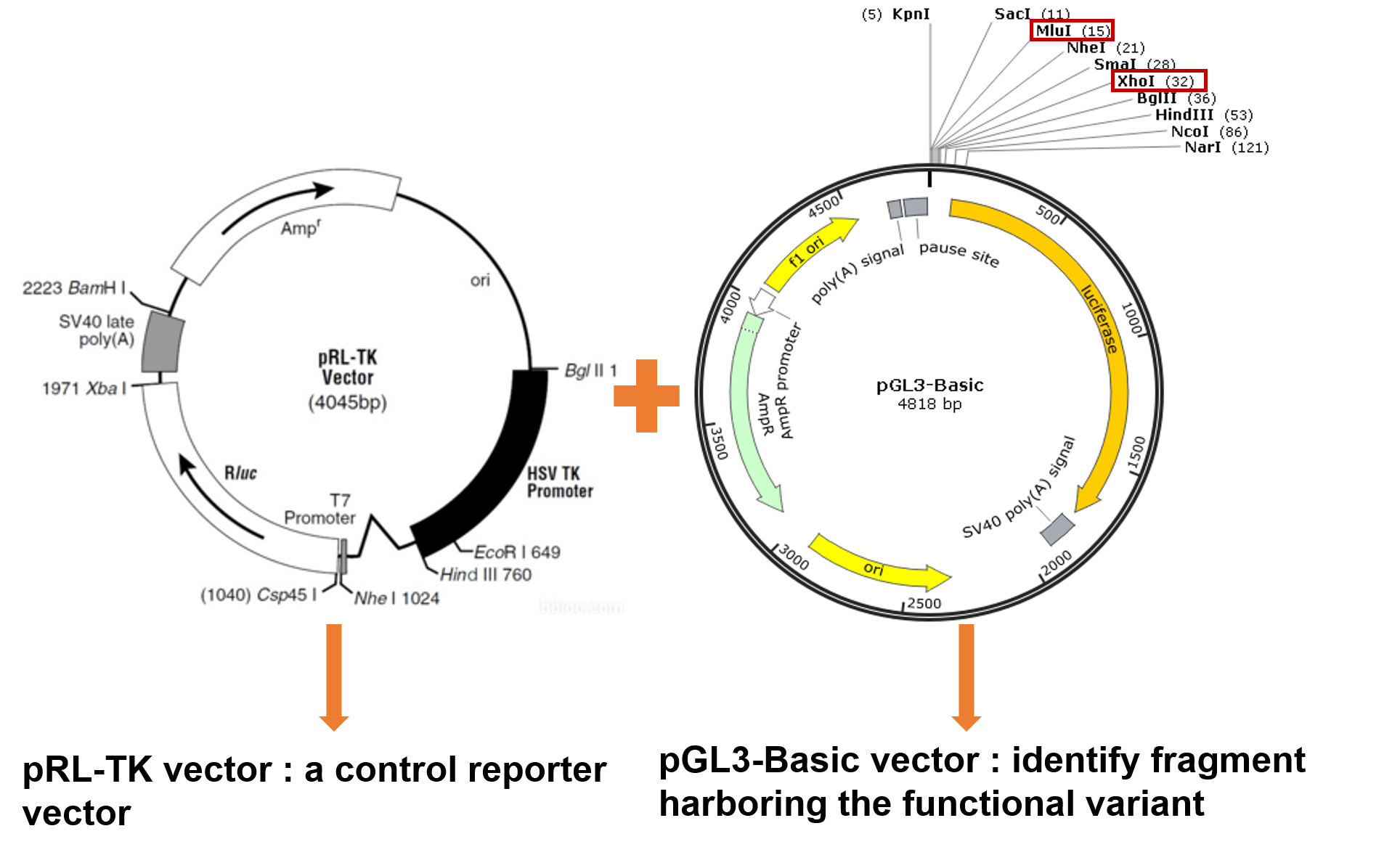


**Figure S13.** The diagram of luciferase reporters used in this study. The red boxes represent the restriction sites of *DGAT1*.
